# Lactoferrin deficiency during lactation increases the risk of depressive-like behavior in adult mice

**DOI:** 10.1186/s12915-023-01748-2

**Published:** 2023-10-31

**Authors:** Wenli Wang, Zhimei Cheng, Xiong Wang, Qin An, Kunlun Huang, Yunping Dai, Qingyong Meng, Yali Zhang

**Affiliations:** 1 College of Food Science and Nutritional Engineering, China Agricultural University, Beijing, China; 2 College of Biological Sciences, China Agricultural University, Beijing, China

**Keywords:** Lactation, Lactoferrin, Depression, Microbial–intestinal–brain, Innate immunity, Inflammation

## Abstract

**Background:**

Lactoferrin is an active protein in breast milk that plays an important role in the growth and development of infants and is implicated as a neuroprotective agent. The incidence of depression is currently increasing, and it is unclear whether the lack of lactoferrin during lactation affects the incidence of depressive-like behavior in adulthood.

**Results:**

Lack of lactoferrin feeding during lactation affected the barrier and innate immune functions of the intestine, disrupted the intestinal microflora, and led to neuroimmune dysfunction and neurodevelopmental delay in the hippocampus. When exposed to external stimulation, adult lactoferrin feeding-deficient mice presented with worse depression-like symptoms; the mechanisms involved were activation of the LPS–TLR4 signalling pathway in the intestine and hippocampus, reduced BDNF-CREB signaling pathway in hippocampus, increased abundance of depression-related bacteria, and decreased abundance of beneficial bacteria.

**Conclusions:**

Overall, our findings reveal that lactoferrin feeding deficient during lactation can increase the risk of depressive-like behavior in adults. The mechanism is related to the regulatory effect of lactoferrin on the development of the "microbial–intestinal–brain" axis.

**Supplementary Information:**

The online version contains supplementary material available at 10.1186/s12915-023-01748-2.

## Background

Depressive disorders are a global public health concern and a leading cause of disease burden, substantially impairing patients’ quality of life and increasing mortality risk. According to the World Health Organization (WHO), the number of people affected by depression increased by 18% worldwide in the decade from 2005 to 2015. In the first year of the COVID-19 pandemic, the global prevalence of anxiety and depression increased by a massive 25%, according to a scientific brief released by the WHO. Depression is caused by complex interactions of multiple factors. The pathogenesis is not fully understood, although many hypotheses have been proposed, such as the inflammatory hypothesis [1], “leaky gut” hypothesis [2], gut microbiota hypothesis [3], and monoamine hypothesis [4]. Biochemical signals that occur between the gastrointestinal tract and central nervous system are known as the gut brain axis. The bidirectional communication between the brain and gut occurs through the autonomic, enteric, neuroendocrine, and immune systems [3]. Studies of the microbiota–gut–brain axis may lead to novel approaches for preventing and treating mental illnesses, including anxiety and depression.

Immune system disorders caused by impaired gastrointestinal barrier integrity are considered as important mechanisms of depression [5]. The intestinal epithelial barrier serves as the first boundary of defence between the organism and luminal environment [6]. Increased intestinal permeability caused by intestinal barrier injury leads to systemic chronic inflammation, whereby nervous system inflammation is an important pathological feature of depression. Lipopolysaccharide (LPS) from bacteria in the intestinal lumen, mainly gram-negative bacteria, enters the systemic circulation through the increased intercellular space and cross-cellular pathways [7] and, LPS binds to TLR-4 in the central nervous system, thus triggering neuroinflammation [8]. Depression is a common comorbidity associated with gastrointestinal diseases, such as irritable bowel syndrome and inflammatory bowel diseases (IBDs) [9]. A previous study showed that chronic gastrointestinal inflammation induces anxiety-like behaviour and alters the central nervous system biochemistry [10]. A population- based study revealed that individuals with IBD had a higher prevalence of depression than matched controls [11]. Gampierakis et al. showed that acute and chronic experimental colitis affects adult hippocampal neurogenesis and innate immune cell responses, which may be mechanisms of cognitive and mood dysfunction in patients with IBD [12].

The gut microbiota is a key regulator of the gut–brain axis and influences brain function and behaviour through the microbiota–gut–brain axis. Many antibodies against intestinal symbiotic bacteria LPS have been detected in the serum of patients with depression, supporting the notion that inflammation is related to intestinal symbiotic bacteria [2]. Animal experiments add further support to this idea; interestingly, faecal microbiota transplantation from depressed patients to microbiota-depleted rats induced anxiety and depressive behaviours and altered tryptophan metabolism in recipient animals [13]. Furthermore, bacterial species regulate the production of neurotransmitters and their precursors, such as serotonin, γ-aminobutyric acid type A (GABA), and tryptophan [14]. Gut microbes also promote the release of short-chain fatty acids (SCFAs) and brain-derived nutritional factors (BDNFs), which have been shown to ameliorate depression [10].

Lactation is a critical period for the development of the intestinal tract and nervous system and the establishment of the gut microbiota; breast milk plays important roles in these processes. However, many newborns cannot be breastfed due to the nutritional and health status of their mothers, social factors, and uncertain circumstances. In low- and middle-income countries, only 37% of children younger than six months of age are exclusively breastfed; this rate is even lower in high-income countries [15]. Lactoferrin (LF) is an important active whey protein in breast milk present at concentrations of 1–7 and 0.2–1.5 g/L in humans and bovines, respectively [16]. LF plays an important role in neonatal development by promoting maturation of the neonatal small intestine and increasing the integrity of the intestinal barrier [17]. Several studies have shown that LF can improve neural development, neuroprotection, and cognitive abilities [18]. The early gut microbiota has been predicted to contribute to disease progression later in life, and the foundation for a stable adult gut microbiota has already been established in infancy [19]. According to a previous study, early life LF intervention can modulate the microbial community of the cecum [20]; however, whether the effects of LF on the microbiota of suckling mice affect adult depression remains unclear. Formula is an important alternative to breast milk, but the addition of LF to formula is not widespread. Thus, considering that exclusive breastfeeding rates are < 50% in most countries, LF deficiency during lactation may be prevalent worldwide. The effect of LF feeding deficiency during lactation on adult health, particularly depression, remains unknown.

In this study, we generated an *Ltf*-knockout (KO) mouse model as a mother mouse that provided LF-free milk. Two groups of mice were evaluated: wild-type (WT) neonates breast-fed by WT or KO female mice (WT-WT or KO-WT). The chronic unpredictable mild stress (CUMS) depression model was established when the mice reached 9 weeks of age, and differences in depressive phenotypes were compared among groups. To investigate the mechanism by which LF feeding deficiency during lactation increases the risk of depressive-like behavior in adult mice, the intestinal and brain development and intestinal microbe composition at different growth stages of mice were investigated.

## Results

### LF feeding deficiency during suckling period increased depressive-like behavior risk in adult mice

**Fig. 1:**
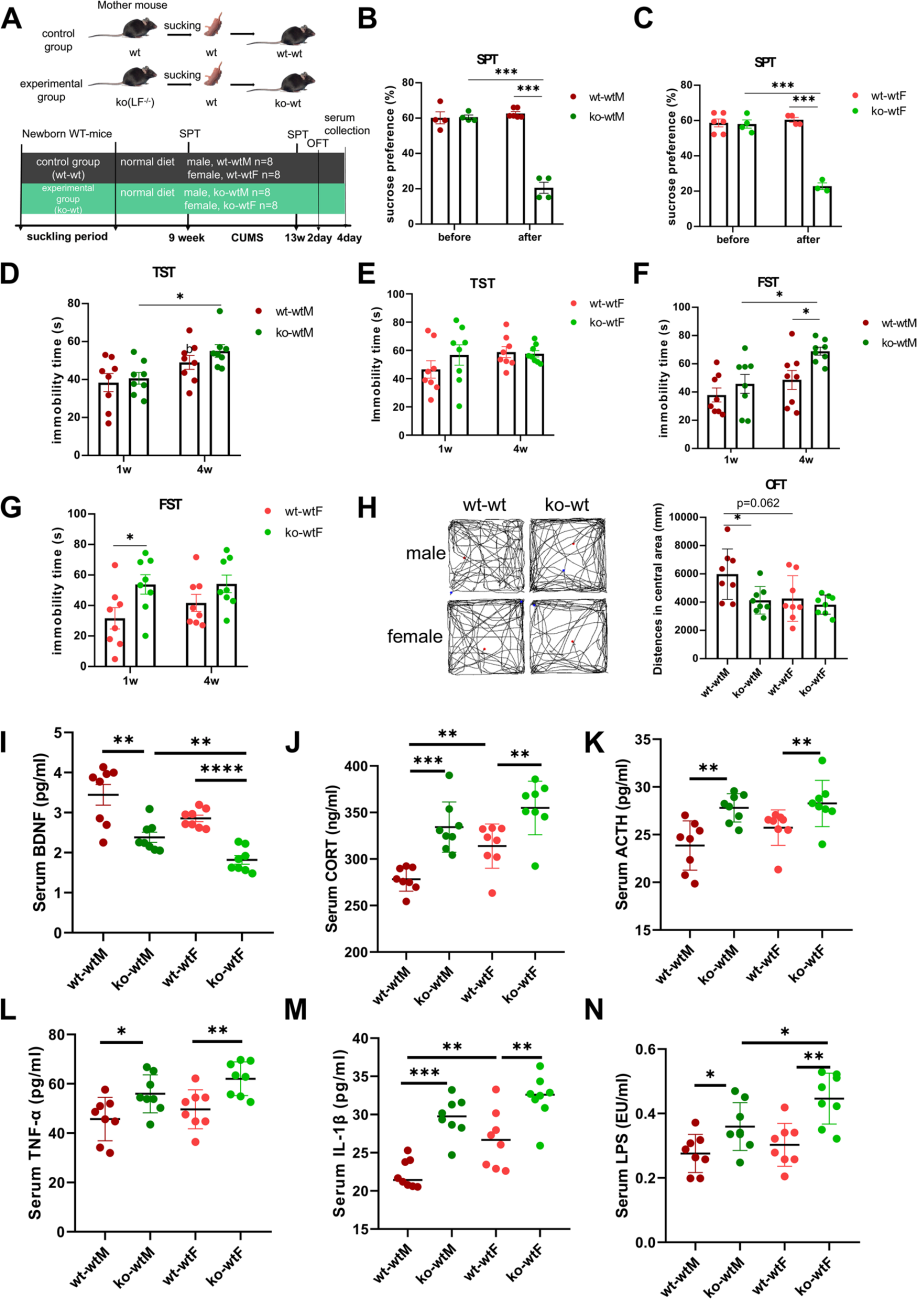
LF feeding deficiency during suckling period increased depressive-like behaviors of CUMS mice in adult. **A** Study design of mice experiment. **B** Percent of sucrose consumption in SPT of male mice. *n* = 4–6. **C** Percent of sucrose consumption in SPT of female mice. *n* = 3–6. **D** Immobility time in the TST of male mice. **E** Immobility time in the TST of female mice. **F** Immobility time in the FST of male mice. **G** Immobility time in the FST of female mice. **H** Trajectory map and distance in central area in OFT of male and female mice. **I** Serum levels of brain-derived neurotrophic factor (BDNF), **J** corticosterone (CORT), **K** adrenocorticotropic hormone (ACTH), **L** tumor necrosis factor-α (TNF-α). **M** interleukin-1β (IL-1β), **N** lipopolysaccharide (LPS). **D**-**N** *n* = 8. Data are presented as mean ± SEM. Two-way ANOVA with multiple comparisons and two-tailed t test for normally distributed data, two-tailed Mann–Whitney test for non-normally distributed data, *P < 0.05; **P < 0.01; ***P < 0.001

Male and female mice that consumed normal milk and LF-free milk during the suckling period were fed a normal diet until 9 weeks of age, then SPT was tested before CUMS, after which, the CUMS model was established for 4 weeks, then SPT was tested again after CUMS, OFT was carried out on the second day after CUMS, serum was collected on the fourth day after CUMS (Fig. 1A). As shown in Fig. 1B (F = 1.820, df = 6, *p* = 0.919) and 1C (F = 0.298, df = 8, *p* = 0.857), wt-wt and ko-wt mice did not differ before CUMS; however, after 4 weeks of CUMS treatment, mice without LF intake during the suckling period (ko-wtM and ko-wtF) showed a lower sucrose preference in the SPT (Mann–Whitney U tests, Fig. 1B: *p* = 0.009 < 0.05; Fig. 1C: *p* = 0.031 < 0.05). In LF-feeding mice the sucrose preference before CUMS has no difference with after in both male and female mice (Fig. 1B: F = 2.146, df = 8, *p* = 0.429; Fig. 1C: F = 1.916, df = 8, *p* = 0.575), but the difference was significant in LF-feeding deficient mice (Mann–Whitney U tests, Fig. 1B: *p* = 0.019 < 0.05; t test, Fig. 1C: F = 0.259, df = 5, *p* = 0.000 < 0.05).

In the TST (Fig. 1D), there was no significant difference between the wt-wtM and ko-wtM groups during the first week of CUMS (F = 2.39, df = 14, *p* = 0.696). At week 4, the immobility time of the wt-wtM (F = 1.128, df = 14, *p* = 0.099 > 0.05) and ko-wtM (Mann–Whitney U tests, *p* = 0.017 < 0.05) groups was higher than that of week 1 (wt-wtM). Although a similar trend was observed in the female group (Fig. 1E), no significant difference was observed between the wt-wtF and ko-wtF groups at weeks 1 (F = 0.382, df = 14, *p* = 0.297) and week 4 (F = 1.75, df = 14, *p* = 0.798). In the FST (Fig. 1F), there was no difference between wt-wtM and ko-wtM mice at week 1 (F = 0.796, df = 14, *p* = 0.362); at week 4, the immobility time of the wt-wtM (F = 0.445, df = 14, *p* = 0.22) and ko-wtM (F = 6.571, df = 9.342, *p* = 0.011 < 0.05) groups increased compared with that of week 1, and the immobility time of the ko-wtM mice was longer than that of the wt-wtM mice (F = 4.69, df = 9.337, *p* = 0.02 < 0.05). Figure 1G shows that the immobility time in the FST of the ko-wt female mice (ko-wtF) was significantly longer than that of the wt-wtF group at week 1 (F = 0.207, df = 14, *p* = 0.034 < 0.05), and increased compared with that of the wt-wtF mice at week 4, but the difference was not significant (F = 0.028, df = 14, *p* = 0.14). What’s more, there were no interaction effects between time and group (Fig. 1D-G). In the OFT, the distance in the central area for the ko-wtM group was significantly smaller than that for the wt-wtM group (F = 2.942, df = 14, *p* = 0.022 < 0.05), with no difference between the ko-wtF and wt-wtF groups (F = 5.474, df = 9.393, *p* = 0.501 > 0.05) (Fig. 1H). The influence of lactoferrin intake during lactation on OFT is significant (F (1,28) = 5.801, *p* = 0.023 < 0.05). Male mice with LF feeding deficiency during lactation showed more severe anxiety-like behaviours in the OFT compared to male mice that drank normal milk. The influence of gender on open field is also significant (F (1,28) = 4.586, *p* = 0.041 < 0.05). Figure 1H shows that the distance in the central area for the wt-wtF group was lower than that for the wt-wtM group (F = 0.062, df = 14, *p* = 0.062 > 0.05), indicating that female mice may exhibited more severe anxiety-like behaviours compared to male mice.

Serum indicators associated with depression were measured. BDNF is a well-known growth factor in the brain [21], and the level of BDNF in the ko-wt group was significantly lower than that in the wt-wt group, for both male and female mice (ko-wtM vs. wt-wtM (F = 9.376, df = 10.191, *p* = 0.004); ko-wtF vs. wt-wtF (F = 0.626, df = 14, *p* = 0.000 < 0.05)). The levels of BDNF in female mice were lower than those in male mice (wt-wtF vs. wt-wtM (F = 19.838, df = 8.369, *p* = 0.059 > 0.05); ko-wtF vs. ko-wtM (F = 0.215, df = 14, *p* = 0.004 < 0.05)) (Fig. 1I). Corticosterone (CORT) and adrenocorticotropic hormone (ACTH) are hormones related to the hypothalamic–pituitary–adrenal axis and are associated with depression. Our results revealed a significantly increased production of CORT and ACTH in the ko-wt mice compared to that in the wt-wt mice (ko-wtM vs. wt-wtM (F = 1.945, df = 14, *p* = 0.00 < 0.05), (F = 3.67, df = 14, *p* = 0.002 < 0.05); ko-wtF vs. wt-wtF(Mann–Whitney U tests, *p* = 0.009 < 0.05, *p* = 0.009 < 0.05) (Fig. 1J, K); the wt-wtF group showed significantly higher CORT levels than the wt-wtM group (Mann–Whitney Test, *p* = 0.009 < 0.05). The levels of pro-inflammatory cytokines, such as tumour necrosis factor (TNF)-α (Fig. 1L) and interleukin (IL)-1β (Fig. 1M), in the ko-wt group were significantly higher than those in the wt-wt group (ko-wtM vs. wt-wtM (F = 0.07, df = 14, *p* = 0.027 < 0.05), (F = 0.266, df = 14, *p* = 0.000 < 0.05); ko-wtF vs. wt-wtF(F = 0.086, df = 14, *p* = 0.005 < 0.05), (F = 0.91, df = 14, *p* = 0.006 < 0.05)), and IL-1β levels in the wt-wtF group were significantly higher than those in the wt-wtM group (F = 3.173, df = 14, *p* = 0.009 < 0.05). Additionally, LPS content was significantly increased in the ko-wt mice serum (ko-wtM vs. wt-wtM (F = 0.446, df = 14, *p* = 0.026); ko-wtF vs. wt-wtF (F = 0.425, df = 14, *p* = 0.002 < 0.05) (Fig. 1N), which can induce inflammation. Furthermore, the LPS levels in the ko-wtF group were significantly higher than those in the ko-wtM group (F = 0.111, df = 14, *p* = 0.04 < 0.05). What’s more, there were no interaction effects between gender and group (Fig. 1H-N).

### CUMS mice with lactation LF feeding deficiency showed intestinal, brain and microbial flora disorders

**Fig. 2:**
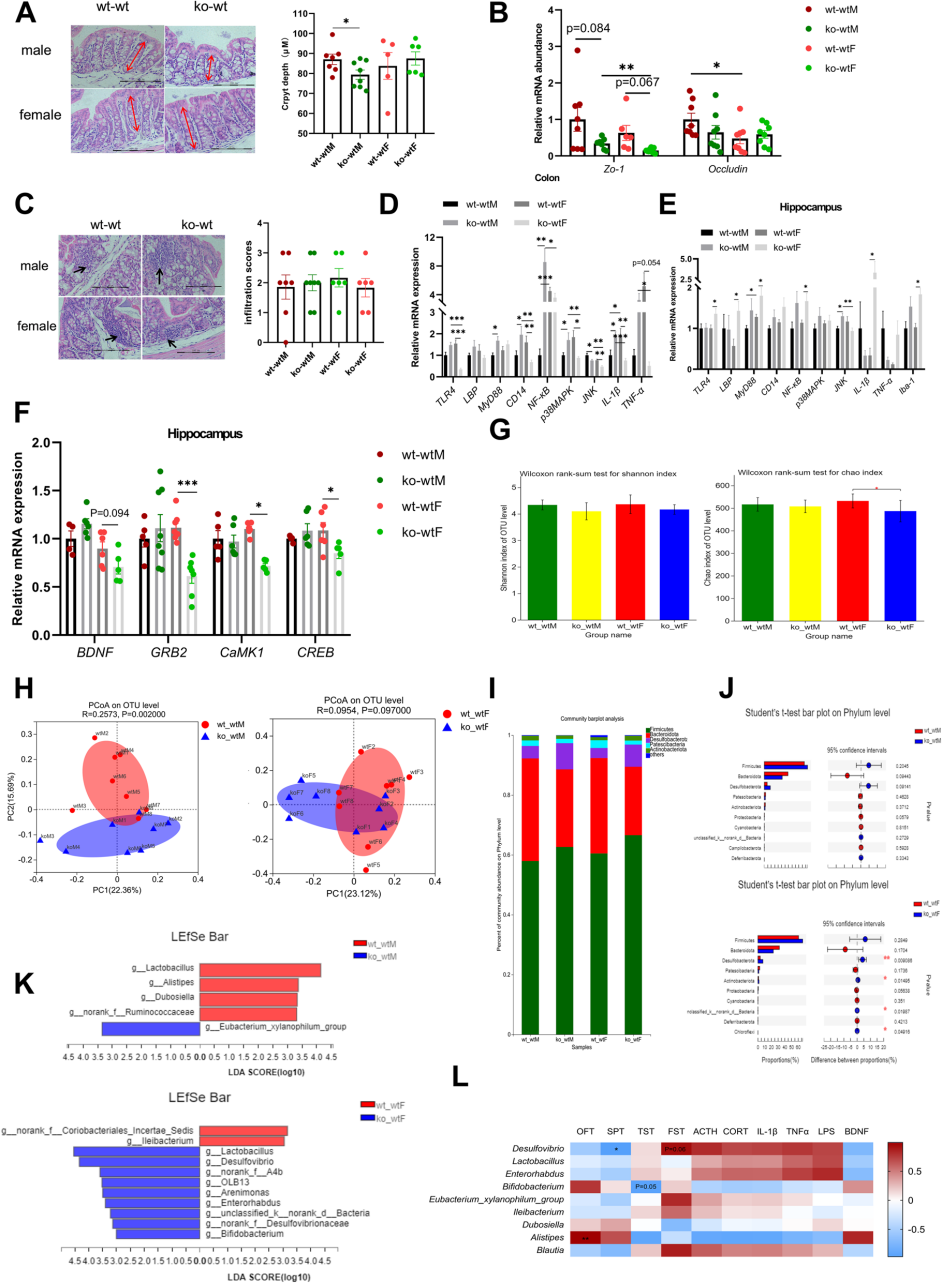
The damage of colon, hippocampus and microorganism composition in lactation LF feeding deficient mice after CUMS model. **A** Crypt depth in the colon of mice and representative images of H.E-stained colonic sections, 200 × , scale bar = 100 μm. *n* = 5–8. **B** *Zo-1*, *Occludin* mRNA expression in the colon. *n* = 6–8. **C** Inflammation infiltration score and representative images in the colon, 200 × , scale bar = 100 μm. *n* = 6–8. **D** Activation of LPS-TLR4 signaling pathway in the colon. *n* = 6–8. **E** Activation of LPS-TLR4 signaling pathway and microglia in the hippocampus. *n* = 6–8. **F** The mRNA expression of *BDNF* signaling pathway in the hippocampus. *n* = 4–8. **G** The alpha diversity analysis, including Shannon diversity and Chao diversity, Wilcoxon rank-sum test. *n* = 8. **H** PCoA analysis results of male and female CUMS mice and evaluated using Analysis of Similarities (ANOSIM). *n* = 8. **I** Analysis on the composition of microbial community at phylum level. *n* = 8. **J** Student’s t-test on Phylum level of male and female depressive-like behavior mice. *n* = 8. **K** Linear discriminant analysis (LDA > 3) scores derived from LEfSe analysis at genus level of male and female depressive-like behavior mice. *n* = 8. **L** Heatmap of Spearman’s correlation between gut microbiota (at the genus level) and depressive-like behavior related indices. *n* = 8. Data are presented as mean ± standard error. **A**-**F **Two-way ANOVA with multiple comparisons and two-tailed t test for normally distributed data, two-tailed Mann–Whitney test for non-normally distributed data, *P < 0.05; **P < 0.01; ***P < 0.001

Dysfunction of the microbiota–gut–brain axis is thought to be the main pathological basis of depression. We compared the histological changes in the colon, inflammation of the colon and hippocampus and composition of intestinal microorganisms in depressed mice with LF feeding deficiency during lactation with those in depressed mice that consumed normal mouse milk during lactation. As shown in Fig. 2A, the crypt depth of ko-wtM mice was significantly lower than that of wt-wtM mice (F = 0.202, df = 13, *p* = 0.046 < 0.05), there was no significant difference in colonic crypt depth between the wt-wtF and ko-wtF groups (F = 1.881, df = 9, *p* = 0.612 > 0.05). Damage to the intestinal epithelial barrier increased the translocation of this luminal LPS to systemic circulation, and expression of the zonula occludens gene (*Zo-1*) in ko-wt mice has no significant difference with wt-wt mice (ko-wtM vs wt-wtM, F = 7.672, df = 7.323, *p* = 0.084 > 0.05; ko-wtF vs wt-wtF, F = 6.858, df = 5.12, *p* = 0.067 > 0.05). However, the results of two-way ANOVA analysis showed that the expression level of *Zo-1* was significantly affected by lactoferrin intake during lactation (F (1,28) = 6.76, *p* = 0.015 < 0.05), *Zo-1* expression in ko-wtF group was significantly lower than that in ko-wtM (F = 2.265, df = 14, *p* = 0.003 < 0.05). There was no significant difference in *Occludin* expression between ko-wtM mice and wt-wtM mice (F = 0.03, df = 14, *p* = 0.188 > 0.05) (Fig. 2B). *Occludin* expression in wt-wtF was significantly lower than that in wt-wtM (F = 1.12, df = 14, *p* = 0.035 < 0.05). Therefore, the intestinal barrier function was more severely damaged in female mice than in male mice, which may be the reason for the higher LPS concentration in ko-wtF mice compared with ko-wtM (Fig. 1N). However, our results showed that the expression of *ZO-1* and *Occludin* in LF lacking mice has no difference with LF feeding mice (ko-wtF vs wt-wtF and ko-wtM vs wt-wtM). It is well known that the increase in serum LPS level is associated with intestinal microflora disorder and intestinal mucosal permeability [22]. Therefore, intestinal microflora disorder may be primarily responsible for the increase of LPS.

As shown in Fig. 2C, the infiltration score of colons showed that no significant difference was found between LF-lacking mice and LF-feeding mice (ko-wtM vs wt-wtM, ko-wtF vs wt-wtF). We further examined the activation of the LPS-TLR4 signaling pathway in the colon and hippocampus. In the colon (Fig. 2D) of male mice, the expression of *Myd88* (*p* = 0.01 < 0.05), *CD14* (F = 0.506, df = 14, *p* = 0.045 < 0.05), nuclear factor *(NF)-κB* (*p* = 0.001 < 0.05), *P38MAPK* (*p* = 0.027 < 0.05) and *IL-1β* (F = 0.921, df = 14, *p* = 0.01 < 0.05) was significantly higher in ko-wtM group than in the wt-wtM group and expression of *TLR4* was higher in the ko-wtM group than in wt-wtM group, but the difference was not significant (*P* = 0.059 > 0.05). *JNK* expression in the wt-wtM group was significantly higher than that in the ko-wtM group (F = 3.4, df = 14, *p* = 0.038 < 0.05); other genes showed no significant differences between these two groups. In female mice, the expression of *TLR-4* (F = 3.834, df = 14, *p* = 0.000 < 0.05), *CD14* (F = 5.039, df = 8.566, *p* = 0.016 < 0.05), *p38MAPK* (*p* = 0.027 < 0.05), *JNK* (F = 0.053, df = 14, *p* = 0.001 < 0.05) and *IL-1β* (F = 3.119, df = 14, *p* = 0.005 < 0.05) was significantly lower in ko-wtF group than in the wt-wtF group, *TNF-α* (F = 14.463, df = 5.102, *p* = 0.054 > 0.05) also show a lower trend in ko-wtF group than in the wt-wtF group. In LF-feeding mice, the expression of *NF-κB* (*p* = 0.001 < 0.05) and *IL-1β* (F = 0.103, df = 14, *p* = 0.045 < 0.05) in female mice was siginificantly higher than male mice. However, in LF-free mice, the expression of *TLR4* (F = 6.524, df = 9.16, *p* = 0.000 < 0.05), *CD14* (F = 3.006, df = 14, *p* = 0.006 < 0.05), *NF-κB* (F = 24.853, df = 8.439, *p* = 0.037 < 0.05), *p38MAPK* (*p* = 0.006 < 0.05), *JNK* (F = 0.017, df = 14, *p* = 0.001 < 0.05), *IL-1β* (F = 1.656, df = 14, *p* = 0.000 < 0.05) and *TNF-α* (*p* = 0.013 < 0.05) in female mice was siginificantly lower than male mice. In the hippocampus (Fig. 2E), the expression of JNK was significantly higher in the ko-wtM group than in the wt-wtM group (F = 1.738, df = 11, *p* = 0.017 < 0.05). The expression of *TLR-4* (F = 1.93, df = 13, *p* = 0.031 < 0.05), *LBP* (F = 1.601, df = 13, *p* = 0.011 < 0.05), *Myd88* (F = 14.221, df = 6.098, *p* = 0.048 < 0.05), *NFκB* (F = 3.946, df = 13, *p* = 0.019 < 0.05) and *IL-1β* (*p* = 0.019 < 0.05) was significantly higher in ko-wtF mice than in wt-wtF mice. The expression of *Iba-1* (a marker of activation for microglia) was significantly higher in ko-wtF mice than in wt-wtF mice (F = 9.926, df = 6.586, *p* = 0.018 < 0.05). In LF-feeding mice, the expression of *Myd88* (F = 0.115, df = 11, *p* = 0.017 < 0.05) in female mice was siginificantly higher than male mice. In LF-free mice, the expression of *JNK* (*p* = 0.008) in female mice was siginificantly lower than male mice.

Thus, after 4 weeks of CUMS, the colon of LF-free male mice showed more severe inflammation which occurred through activation of the *TLR4-Myd88-NF-κB* signaling pathway. However, female mice that consumed normal mouse milk showed more severe intestinal inflammation via activation of the *MAPK* and *JNK* signals in the *TLR4* signaling pathway. The difference in colonic inflammation between female and male mice may be related to different levels of oestrogen [23]. Based on the significantly different levels of BDNF in the serum, we measured the expression of genes related to the *BDNF* signaling pathway in the hippocampus of depressed mice. None of the genes differed between the ko-wtM and wt-wtM group (Fig. 2F); however, in the female groups, the expression of *Grb2* (F = 0.915, df = 13, *p* = 0.000 < 0.05), *CaMK1* (F = 1.652, df = 9, *p* = 0.012 < 0.05), and *Creb* (F = 2.283, df = 9, *p* = 0.048 < 0.05) in the ko-wtF group was significantly lower than that in the wt-wtF group, and the expression of *BDNF* in the ko-wtF mice was lower than that in the wt-wtF group but not significantly (F = 0.346, df = 9, *p* = 0.094 > 0.05). What’s more, gender and group (LF intake or not during lactation) had an significant interactive effect on the expression of *BDNF* (F (1,17) = 6.267, *p* = 0.023 < 0.05)*, GRB2* (F (1,24) = 9.39, *P* = 0.005 < 0.05) and *CREB* ((F(1,17) = 5.394, *p* = 0.033 < 0.05)*.*

Next, we examined the composition of the gut flora. Alpha diversity represents the richness and diversity of the microbial community. The Shannon formula was used to estimate diversity, and the richness of the microbial community was estimated using the Chao index. Mice without LF intake during the suckling period (ko-wtM and ko-wtF) showed a lower Shannon index of the intestinal microbiota but the difference was not significant (Fig. 2G). The Chao index was significantly lower in the ko-wtF than in the wt-wtF mice, but there was no significant difference between the ko-wtM and wt-wtM groups (Fig. 2G). Principal coordinate analysis (PCoA) revealed major differences between the ko-wtM and wt-wtM mice (*p* = 0.002 < 0.05) as well as differences in the microbial composition between the ko-wtF and wt-wtF mice (Fig. 2H, *P* = 0.097 > 0.05). There was a significant difference in the pattern of intestinal flora between the ko-wt-depressed and wt-wt-depressed mice. The predominant phyla (Fig. 2I) were Firmicutes, Bacteroidetes, Desulfobacterota, Patescibacteria, and Actinobacteria in the faecal samples of the four groups, and there was no difference between ko-wtM and wt-wtM mice. In contrast, the abundances of Desulfobacterota, Actinobacteria, and Chloroflexi significantly increased in the ko-wtF group (Fig. 2J). At the genus level, we used linear discriminant analysis effect size (LEfSe) to identify bacteria whose relative abundance significantly differed between ko-wt and wt-wt mice. Figure 2K shows that there was a greater abundance of *Eubacterium_xylanophilum_group,* and a lower abundance of *Lactobacillus* and SCFA-producing bacteria (*Alistipes* and *Dubosiella*) in the ko-wtM group than in the wt-wtM group. SCFAs exert anti-inflammatory functions and are beneficial to the composition of the gut microbiota and intestinal barrier [24]. Figure 2K shows that *Lactobacillus*, *Desulfovibrio*, *Arenimonas*, *Enterorhabdus*, and *Bifidobacterium* were significantly enriched in the ko-wtF group, and *Ileibacterium* was significantly enriched in the wt-wtF group.

To further explore the correlation between the gut microbiota (significantly different microbiota at the genus level) and depression-related behavioural indices (OFT, SPT, TST, and FST), depression-related hormones (ACTH and CORT), serum inflammatory factors (IL-1β and TNF-α), and BDNF, a heatmap of Spearman’s correlation analysis was generated (R and *P* value was showed in Additional file: Table. S1, S2). As shown in Fig. 2L, *Desulfovibrio* was significantly negatively correlated with SPT and positively correlated with FST (*P* = 0.06); *Bifidobacterium* was negatively correlated with TST (*P* = 0.05); and *Alistipes* was significantly positively correlated with OFT. Furthermore, *Lactobacillus* and *Enterorhabdus* were positively correlated with TST, ACTH, CORT, IL-1β, TNF-α, and LPS, and negatively correlated with OFT, SPT, and BDNF. *Blautia* showed a positive correlation with ACTH, FST, TST, CORT, IL-1β, TNF-α, LPS, and depressive-like behavior.

### Intestinal growth retardation and intestinal microbiological disorder in 18-day-old suckling mice with LF feeding deficiency during lactation

**Fig. 3:**
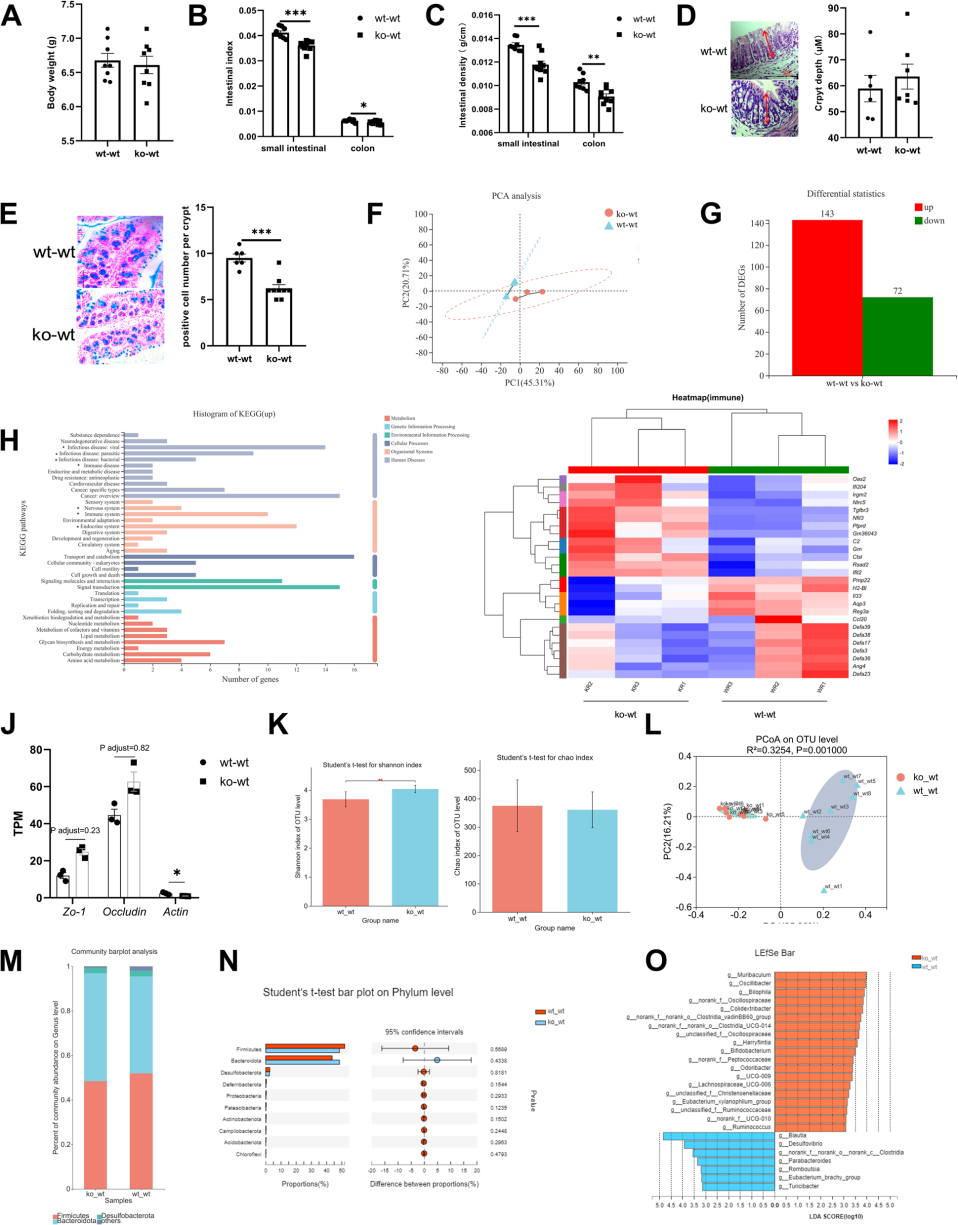
Effects of LF feeding deficiency during lactation on intestinal development and intestinal microorganisms in 18-day-old suckling mice. **A** Body weight of 18-day-old mice. *n* = 8. **B** Small intestine and colon index of 18-day-old mice. *n* = 8–9. **C** Intestinal density of small intestinal and colon of 18-day-old mice. *n* = 8–9. **D** Crypt depth in the colon of 18-day-old mice and representative images of H.E-stained colonic sections, 200 × , scale bar = 100 μm. *n* = 6–8. **E** Counts of the goblet cells in the colon of 18-day-old mice and representative images of AB-stained colonic sections, 200 × , scale bar = 100 μm. *n* = 6–9. (**A**-**E**, data are presented as mean ± standard error. Student's t-test was employed for comparisons between two groups; *P < 0.05; **P < 0.01; ***P < 0.001). **F** Principal components analysis (PCA) of small intestinal RNAseq. **G** Differential expression genes (DEGs) between wt-wt group and ko-wt group mice. (*P* adjust < 0.05, fold change ≥ 2). **H** KEGG functional annotation analysis of differential genes of wt-wt and ko-wt group mice. **I** The clustering heatmap of immune related DEGs between wt-wt group and ko-wt group. **J** Tight junction genes expression in RNAseq. **F**-**J**, *n* = 3. **K** Alpha diversity index difference analysis of the wt-wt and ko-wt group: Shannon index, Chao index. Wilcoxon rank-sum test. **L** Principal coordinates analysis (PCoA) of bacterial beta-diversity based on the Bray–Curtis dissimilarity index and evaluated using ANOSIM. Each symbol represents a single sample of feces. **M** Composition of microbial community at phylum level of 18-day-old sucking mice. **N** Student’s t-test on Phylum level of 18-day-old sucking mice. **O** Linear discriminant analysis (LDA > 3) scores derived from LEfSe analysis at genus level of 18-day-old sucking mice, **K**–**O**, *n* = 8

Our experiments showed that LF intake during lactation significantly affected depressive-like behavior in adult mice, which was induced by differences in body development caused by LF intake during lactation. Therefore, we used 18-day-old mice with almost no feed intake for analysis. The body weight of 18-day-old suckling mice without LF feeding during lactation did not differ from that of control mice (Fig. 3A) but the intestinal index and density of the small intestine (F = 0.145, df = 16, *p* = 0.000 < 0.05; F = 1.282, df = 15, *p* = 0.000 < 0.05) and colon (F = 1.39, df = 16, *p* = 0.031 < 0.05; F = 0.025, df = 15, *p* = 0.002 < 0.05) decreased significantly (Fig. 3B, C). Crypt depth did not differ significantly between the two groups (Fig. 3D). The number of colonic goblet cells in the ko-wt group was significantly lower than that in the control group (F = 0.012, df = 13, *p* = 0.000 < 0.05) (Fig. 3E). There was no significant difference in the number of Paneth cells per crypt in colon between two group (Additional file: Fig. S1).

To further explore the effect of LF feeding deficiency on intestinal development during lactation, we performed genome-wide transcriptional profiling of the small intestine of the two groups of mice for RNA sequencing. PCoA showed that the transcriptomes of ko-wt and wt-wt mice were clearly separated (Fig. 3F). Transcriptome sequencing showed that 143 genes were upregulated and 72 genes downregulated in the small intestine of the ko-wt mice compared to those of the wt-wt mice (fold-change > 1, *P* adjust < 0.05, Fig. 3G). These DEGs were subjected to pathway analysis using the Kyoto Encyclopedia of Genes and Genomes (KEGG) database, which showed that upregulated mRNAs were mainly involved in infectious, immune, and neurodegenerative diseases, and were enriched in the nervous, immune, and endocrine systems (Fig. 3H). We explored immune-related genes in the small intestine using an RNA-seq transcriptomic profiling approach, as shown in Fig. 3I; genes encoding α-defensin, such as *Defa23*, *Defa36*, *Defa3*, *Defa17*, *Defa38*, and *Defa39*, were significantly decreased in the ko-wt group. The RNA-Seq results showed no difference in the expression of the tight junction proteins *zo-1* and *occludin* between the two groups. However, the expression of the gene encoding the actin protein (*Gm12715*) was significantly decreased (Fig. 3J); this gene is related to the assembly of tight junctions.

To determine the effect of LF deficiency during lactation on intestinal microorganisms, we analysed the gut microbiota composition by 16S rRNA gene amplicon sequencing of the caecal contents of 18-day-old mice. Alpha diversity measures (Shannon and Chao diversity indices), calculated for each group, showed that the Chao diversity index of the ko-wt group did not differ from that of the wt-wt group; however, the Shannon index was significantly higher than that of the control group (Fig. 3K). These results indicate that the intestinal microbial alpha diversity was increased in 18-day-old mice not fed LF. PCoA of the beta-diversity comparison revealed significant separation between the two groups of microbial communities (Fig. 3L). At the phylum level, Firmicutes, Bacteroidetes, and Desulfobacterota accounted for the majority of the bacteria (Fig. 3M), with no differences between the two groups (Fig. 3N). At the genus level, the LEfSe results revealed a greater abundance of *Muribaculum*, *Oscillibacter*, *Bilophila*, *Colidextribacter*, *Harryflintia*, *Bifidobacterium*, *Odoribacter*, *UCG-009*, *Lachnospiraceae_UCG-006*, *Eubacterium_xylanophilum_group*, *Ruminococcus*, and some unclassified microbiotas; there was a lower abundance of *Blautia*, *Desulfovibrio*, *Parabacteroides*, *Romboutsia*, *Eubacterium_brachy_group*, and *Turicibacter* in the ko-wt group (Fig. 3O).

### LF feeding deficiency during lactation induced hippocampal growth retardation

**Fig. 4:**
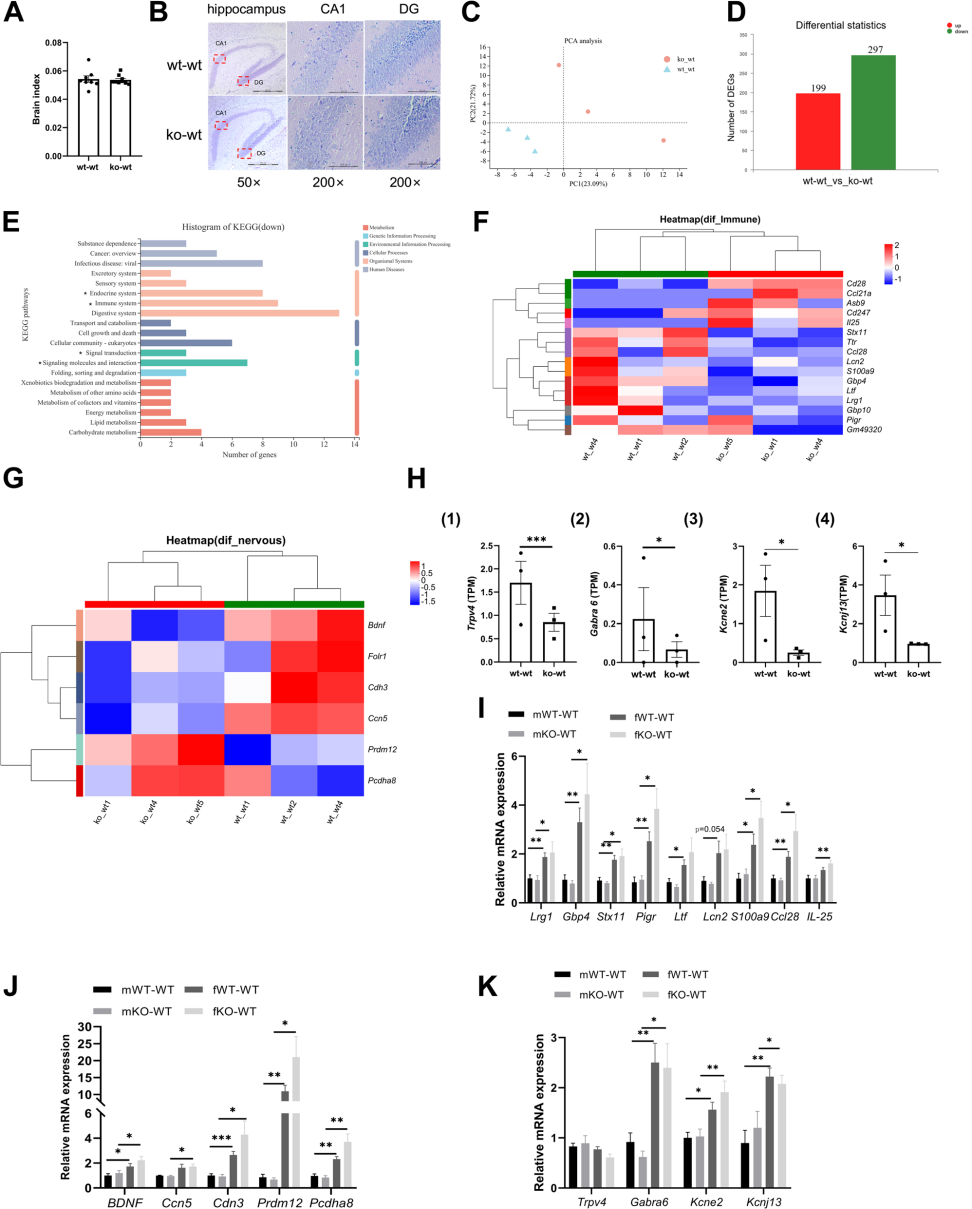
LF feeding deficiency during lactation affects the development of hippocampus in 18-day old and adult mice. **A** The brain index of 18-day-old mice in wt-wt and ko-wt group. **B** The representative H.E staining of hippocampus (50 × , scale bar = 500 μm), hippocampus CA1 and DG region (200 × , scale bar = 100 μm) in 18-day-old mice. **A**-**B,** *n* = 8–9. (C)Principal components analysis (PCA) of hippocampus RNAseq in 18-day-old mice. (D)Differential gene expression between wt-wt group and ko-wt group mice (18-day-old), (*P* value < 0.05, fold change ≥ 2). **E** KEGG functional annotation analysis of differential genes in 18-day-old mice. **F**, **G** The clustering heatmap of immune, nervous development response-related DEGs between wt-wt group and ko-wt group (18-day-old). **H** Expression of nervous signal transduction related genes in 18-day old mice RNAseq. **I** Expression of immune related genes in the hippocampus of adult mice. **J** Expression of nervous development related genes in the hippocampus of adult mice. **K** Expression of nervous signal transduction related genes in the hippocampus of adult mice. **C**-**H**, *n* = 3. (I)-(K), *n* = 7–8, Two-way ANOVA with multiple comparisons and two-tailed t test for normally distributed data, two-tailed Mann–Whitney test for non-normally distributed data, *P < 0.05; **P < 0.01; ***P < 0.001

Considering that depression is a mental illness, it is necessary to explore the effects of LF deficiency during lactation on the hippocampus. Figure 4A shows that there was no significant difference in the brain index between ko-wt and wt-wt 18-day-old mice. The CA1 region of the hippocampus is involved in cognitive processes, learning, and memory [25]. The hippocampal dentate gyrus (DG) is a key target region for both antidepressant effects and stress-related processes [26]. In the wt-wt group, pyramidal cells in the CA1 region were arranged neatly in dense layers and had regular and clear structures. No significant difference was observed between the ko-wt and wt-wt groups in cell morphology in the DG region (Fig. 4B).

RNA-seq of the hippocampi of 18-day-old mice was performed. PCA showed that the transcriptomes of the two groups were clearly distinct (Fig. 4C). Transcriptome sequencing results showed that 199 genes were upregulated and 297 genes downregulated in the hippocampi of the ko-wt mice compared to those of the wt-wt mice (fold-change > 1, *P* < 0.05, Fig. 4D). The KEGG pathway annotation results showed that the downregulated mRNAs mainly participated in signalling molecules and interactions, and signal transduction, and were enriched in the immune system (Fig. 4E). Figure 4F shows that the expression of genes involved in innate immunity, such as *Lrg1*, *Gbp4* [27], *Gbp10* [27], and *Stx11* [28], was decreased. Genes involved in adaptive immunity, such as *Ccl21a* [29], *Cd247* [30], *Cd28* [31], and *IL25* [32], showed increased expression. The gene expression of *Pigr* [33], *Ltf*, *Lcn2* [34], *S100a9* [35], and *Ccl28* [36], which are involved in both innate and adaptive immune responses, decreased. Some genes involved in the development of the nervous system in the ko-wt group were significantly different from those of the wt-wt group. The gene expression of *BDNF*, *Cdh3*, *Ccn5*, and *Folr1* decreased, and that of *Prdm12* and *Pcdha8* increased (Fig. 4G). These differences may lead to the delayed development of the nervous system. Figure 4H shows that the expression of genes related to neuronal signal transduction (*Trpv4*, *Gabra6*, *Kcne2*, and *Kcnj13*) decreased in the ko-wt group.

To explore whether the influence of LF-deficient feeding during lactation on the hippocampus extends into adulthood, we assayed differential gene expression in the hippocampus of adult mice. Figure 4I shows that there was no significant difference between KO-WT mice (mKO-WT, fKO-WT) and WT-WT mice (mWT-WT, fWT-WT) in immune-related differential expression genes (DEGs), but gene expression in female mice was significantly higher than that in male mice. Figure 4J shows that the expression of *BDNF*, *Ccn5*, *Cdn3*, *Prdm12* and *Pcdha8* in KO-WT mice (mKO-WT, fKO-WT) did not differ from that in WT-WT mice (mWT-WT, fWT-WT). However, the effect of sex on these genes is significant. The expression of *BDNF* (F = 0.188, df = 14, *p* = 0.021 < 0.05), *Cdn3* (F = 3.116, df = 14, *p* = 0.000 < 0.05), *Prdm12* (*p* = 0.004 < 0.05) and *Pcdha8* (*p* = 0.004 < 0.05) in fWT-WT group was significantly higher than mWT-WT group. The expression of *BDNF* (F = 0.84, df = 14, *p* = 0.013 < 0.05), *Ccn5* (F = 7.919, df = 5.673, *p* = 0.012 < 0.05), *Cdn3* (F = 56.901, df = 7.29, *p* = 0.018 < 0.05), *Prdm12* (F = 61.522, df = 5.006, *p* = 0.02 < 0.05) and *Pcdha8* (F = 34.899, df = 5.574, *p* = 0.006) in fWT-WT group was significantly higher than mWT-WT group. Figure 4K shows that the expression of *Trpv4*, *Gabra6*, *Kcne2*, and *Kcnj13* in KO-WT mice (mKO-WT, fKO-WT) did not differ from that in WT-WT mice (mWT-WT, fWT-WT), and the expression of *Gabra6*, *Kcne2*, and *Kcnj13* was significantly higher in female mice than in male mice. When the mice reached adulthood, the difference induced by LF feeding deficiency in the hippocampus was disappeared.

### Lactation LF feeding-deficient adult mice still showed some differences in intestinal and intestinal microorganisms compared with the control group

To explore whether the effects of LF feeding deficiency during lactation on intestinal and intestinal microorganisms continue into adulthood, mice were fed normally until adulthood after weaning.

**Fig. 5:**
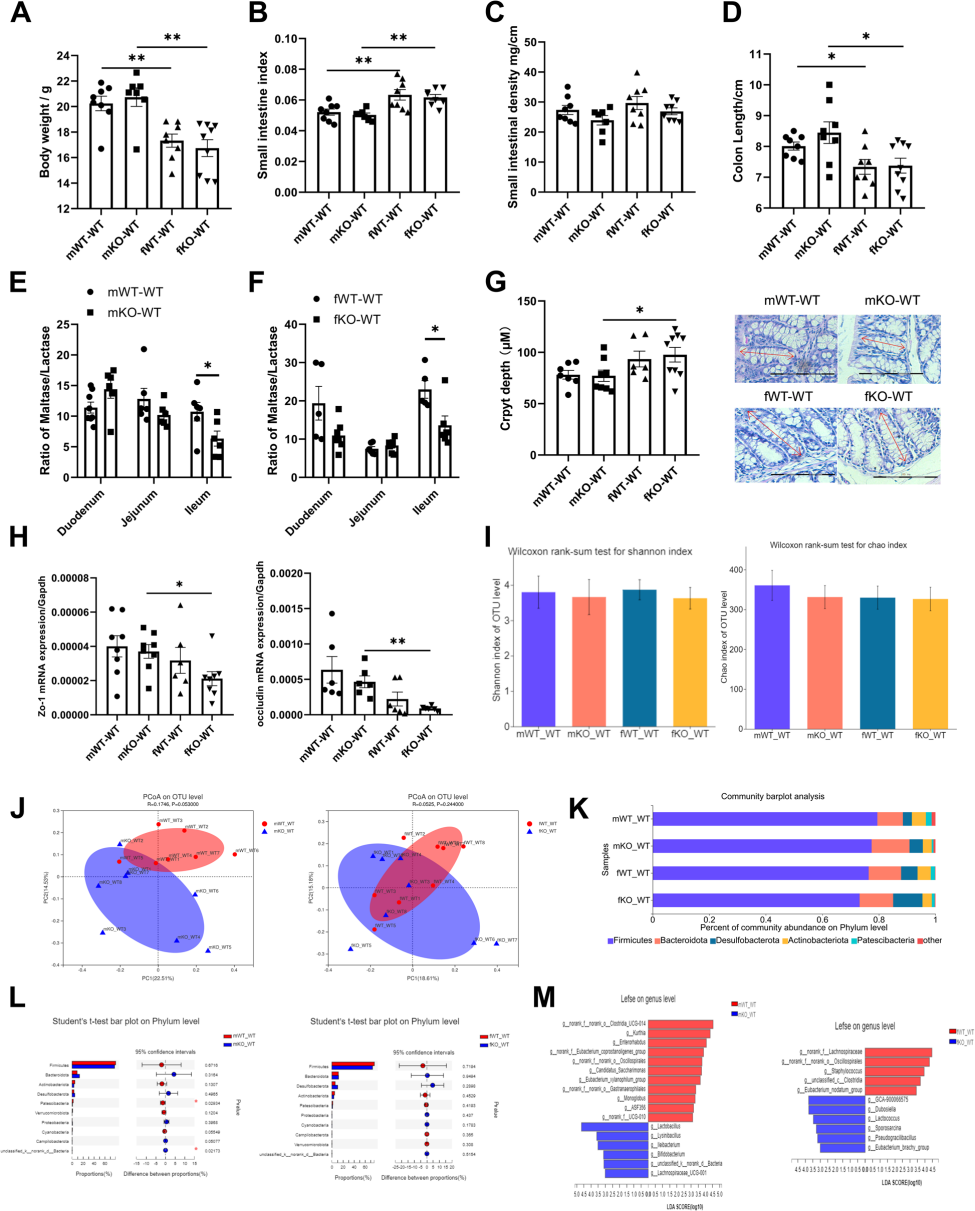
Effects of LF feeding deficiency during lactation on intestinal development and intestinal microorganisms in adult mice. **A** Body weight of four groups of adult mice. *n* = 8. **B** Small intestinal index of four groups of adult mice. *n* = 8. **C** Small intestinal density of four groups of adult mice. *n* = 8. **D** Colon length of four groups of adult mice. *n* = 8. **E** The ratio of maltase/ lactase in the duodenum, jejunum and ileum of male mice. *n* = 6–8. **F** The ratio of maltase/ lactase in the duodenum, jejunum and ileum of female mice. *n* = 5–8. **G** H.E stain colonic tissue sections and crypt depth of four groups of adult mice, 200 × , scale bar = 100 μm. *n* = 6–8. **H** *Zo-1, Occludin* mRNA expression in the colon of four groups of adult mice. *n* = 6–8. **A**-**H** Two-way ANOVA with multiple comparisons and two-tailed t test for normally distributed data, two-tailed Mann–Whitney test for non-normally distributed data, *P < 0.05; **P < 0.01; ***P < 0.001. **I** The alpha diversity analysis of four groups of adult mice, including Shannon diversity and Chao diversity, Wilcoxon rank-sum test. **J** PCoA analysis results of male and female adult mice and evaluated using ANOSIM. **K** The composition of microbial community at phylum level of four groups of adult mice. **L** Student’s t-test on Phylum level between WT-WT and KO-WT of male and female mice. **M** Linear discriminant analysis (LDA > 3) scores derived from LEfSe analysis at genus level of male and female mice. **I**-**M** *n* = 7–8

Intestinal development was evaluated using four groups (male: mWT-WT, mKO-WT; female: fWT-WT, fKO-WT) of 9-week-old mice which was different from that of 18-day-old mice. There were no significant differences in the body weight (Fig. 5A), small intestinal index (Fig. 5B), small intestinal density (Fig. 5C) and colon length (Fig. 5D) between WT-WT and KO-WT mice either male or female. However, the body weight (fWT-WT vs mWT-WT: F = 0.012, df = 14, *p* = 0.002 < 0.05; fKO-WT vs mKO-WT: Mann–Whitney test, *p* = 0.005 < 0.05) and colon length (fWT-WT vs mWT-WT: F = 2.543, df = 14, *p* = 0.027 < 0.05; fKO-WT vs mKO-WT: F = 0.206, df = 15, *p* = 0.021 < 0.05) of female mice were significantly lighter and shorter than male mice (Fig. 5A, D), the small intestinal index of female mice were significantly higher than male mice (fWT-WT vs mWT-WT: F = 0.012, df = 14, *p* = 0.002 < 0.05; fKO-WT vs mKO-WT: F = 1.521, df = 13, *p* = 0.002 < 0.05) (Fig. 5B). The results of two-way ANOVA in Fig. 5C showed that LF intake situation had a certain effect on small intestine density, but the difference was not significant enough (F (1,27) = 3.563, *p* = 0.07 > 0.05).

The maltase/lactase values of the ileum in the mKO-WT and fKO-WT mice were significantly lower than those in the mWT-WT and fWT-WT mice, respectively (Fig. 5E (F = 0.02, df = 10, *p* = 0.049 < 0.05), 5F (Mann–Whitney test, *p* = 0.028 < 0.05)). As shown in Fig. 5G, there was no difference between KO-WT and WT-WT mice in the crypt depth (mKO-WT vs. mWT-WT; fKO-WT vs. fWT-WT). Figure 5H shows that the expression of *Zo-1* and *Occludin* in KO-WT mice has no significant difference with WT-WT mice (mKO-WT vs. mWT-WT; fKO-WT vs. fWT-WT). *Zo-1* and *Occludin* expression was significantly lower in fKO-WT mice than in mKO-WT mice (F = 0.029, df = 14, *p* = 0.015 < 0.05; F = 7.404, df = 5.234, *p* = 0.006 < 0.05). Thus, the influence of LF feeding deficiency on the intestines of normal adult mice was weakened; however, female mice that consumed LF-free milk during lactation may have a greater risk of the intestinal barrier dysfunction compared with male mice in adulthood. In addition, the ileal maturity of LF-deficient mice was lower than that of LF-drinking mice, and we have previously shown that the maturity of the three segments of the small intestine of 18-day old mice drinking LF-free milk was lower than that of normal mice [17].

To examine the effects of different feeding methods during lactation on the composition of intestinal microflora in healthy adult mice (male: mWT-WT, mKO-WT; female: fWT-WT, fKO-WT), the cecal content was investigated using 16S rRNA gene sequencing. Alpha-diversity analysis revealed no difference in the Shannon and Chao indices between WT-WT and KO-WT mice (mKO-WT vs mWT-WT; fKO-WT vs fWT-WT, Fig. 5I). PCoA of the beta-diversity comparison revealed separation between the mKO-WT and mWT-WT groups, but the difference was not significant (*P* = 0.053). PCoA showed no separation among fKO-WT and fWT-WT groups on OUT level (Fig. 5J). These results suggest that there was no difference in alpha and beta diversity between lactating LF-deficient adult mice and normal mice. The phylum level was dominated by Firmicutes, Bacteroidetes, Desulfobacterota, Actinobacteria, and Patescibacteria (Fig. 5K). Patescibacteria was significantly higher in the mWT-WT group, and there was no difference between the fKO-WT and fWT-WT groups at the phylum level (Fig. 5L). At the genus level, the LEfSe results demonstrated a greater abundance of *Lactobacillus*, *Lysinibacillus*, *Ileibacterium*, *Bifidobacterium*, and *Lachnospiraceae_UCG-001*, whereas there was a lower abundance of *Kurthia*, *Enterorhabdus*, *Candidatus*, *Saccharimonas*, *Eubacteriumxylanophilum_group*, *Monoglobus*, and *ASF356* in the mKO-WT group. *Dubosiella*, *Lactococcus*, *Sporosarcina*, *Pseudogracilibacillus*, and *Eubacteriumbrachygroup* were significantly higher in the fKO-WT group; *Staphylococcus* and *Eubacterium_nodatum_group* were significantly higher in the fWT-WT group (Fig. 5M).

### Lactation LF feeding-deficient adult mice showed severe intestinal injury and intestinal microbial disorder based on DSS-induced colitis

Following LF deficiency during lactation, the intestinal development and composition of intestinal microorganisms in 18-day-old mice significantly differed from those of normal mice; however, these differences were covered and became not obvious after 9 weeks of normal feeding under physiological conditions. To examine whether the two types of mice exhibited the same pathological reaction, a 1-week acute DSS model was established in 8-week-old mice.

**Fig. 6:**
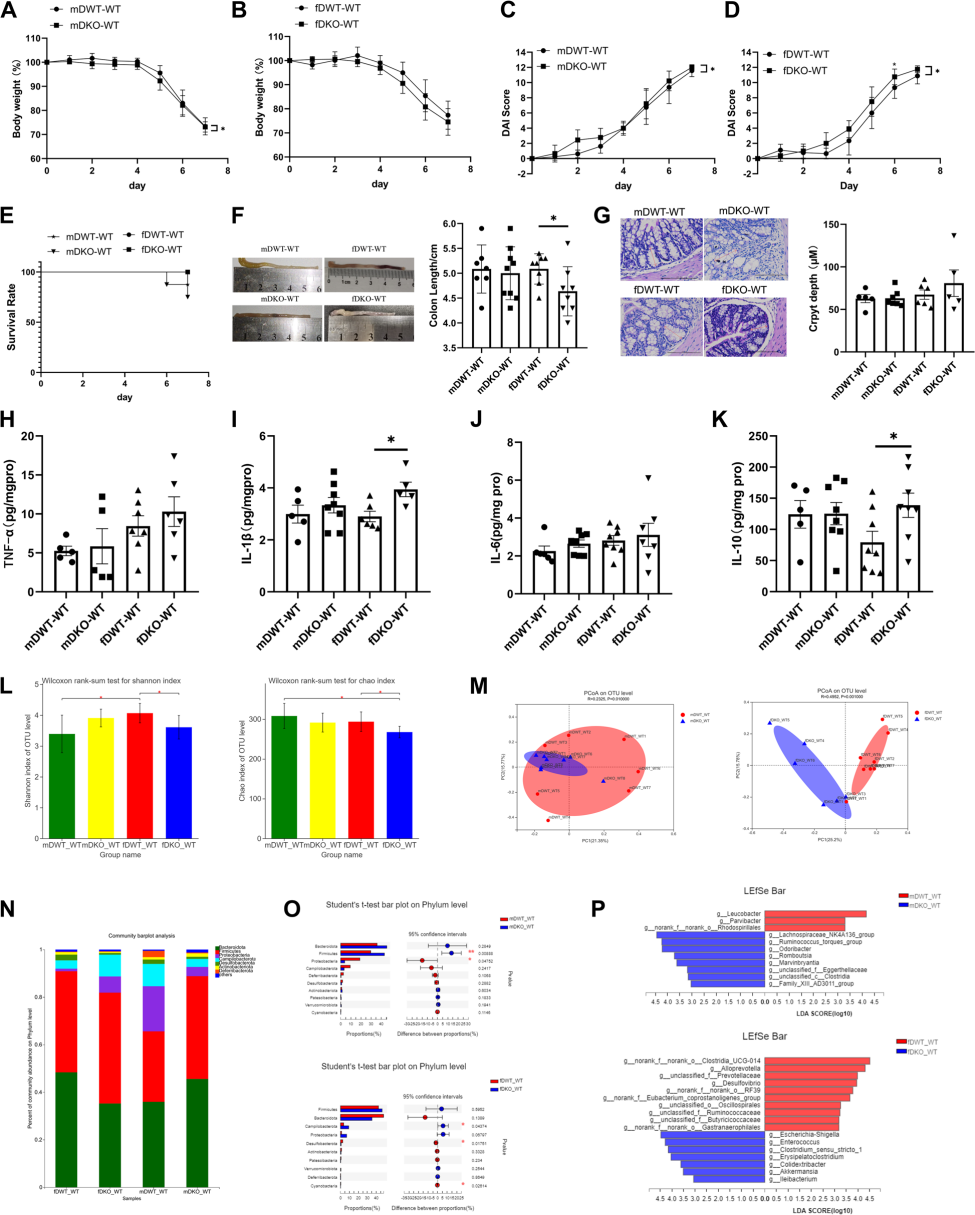
Lactation LF feeding deficiency mice show more severe intestinal damage and gut microbial disorders in DSS model. **A** Changes in body weight of male mice in DSS model within 7 days. **B** Changes in body weight of female mice in DSS model within 7 days. **C** DAI scores of male mice during 7 days of DSS-induced colitis experiments. **D** DAI scores of female mice during 7 days of DSS-induced colitis experiments. **A**-**D** Repeated measures two-way ANOVA test. *n* = 8. **E** Survival rate of mice in DSS model. *n* = 8. **F** Colon length of mice in DSS model. *n* = 7–8. **G** H.E stain colonic tissue sections and crypt depth of DSS model mice, 200 × , scale bar = 100 μm. *n* = 5–8. **H**–**K** The level of TNF-α, IL-1β, IL-6, IL-10 in colon tissue of DSS model mice. *n* = 5–8. Data are presented as mean ± standard error. **F**-**K** Two-way ANOVA with multiple comparisons and two-tailed t test for normally distributed data, two-tailed Mann–Whitney test for non-normally distributed data, *P < 0.05; **P < 0.01; ***P < 0.001. **L** Alpha diversity index difference analysis in DSS model: Shannon index, Chao index. Wilcoxon rank-sum test. **M** Principal coordinates analysis (PCoA) of bacterial β-diversity based on the Bray–Curtis dissimilarity index in DSS model and evaluated using ANOSIM. **N** Composition of microbial community at phylum level in DSS model. **O** Student’s t-test on Phylum level in DSS model. **P** Linear discriminant analysis (LDA > 3) scores derived from LEfSe analysis at genus level. **L**-**P** *n* = 6–8, *P < 0.05; **P < 0.01; ***P < 0.001

In the DSS colitis model mice, the body weight of mDKO-WT mice was significantly lower than mDWT-WT mice (F (1,15) = 7.526, *P* = 0.015 < 0.05) (Fig. 6A). In female mice, the body weights of fDKO-WT mice were consistently lower than those of fDWT-WT mice, but the difference was not significant (F (1,15) = 1.695, *P* = 0.213 > 0.05)), and the body weights of fDWT-WT and fDKO-WT mice decreased to 77.3% and 74.6%, respectively (Fig. 6B). The disease activity index (DAI) score is an effective indicator of colon inflammation and damage. The DAI score of the mDKO-WT group was significantly higher than that of the mDWT-WT group (F (1,15) = 5.873, *p* = 0.028 < 0.05)) (Fig. 6C). The DAI score of the fDKO-WT group was also significantly higher than that of the fDWT-WT group (F (1,15) = 5.668, *p* = 0.031 < 0.05)), and the difference was significant at d 6 and 7 (t test, F = 1.582, df = 15, *p* = 0.034; F = 3.737, df = 15, *p* = 0.05) (Fig. 6D). The survival rate of the mDKO-WT group was lower than that of the mDWT-WT group, and there were no deaths in the two female mouse groups (Fig. 6E). There was no difference in colon length between the two groups of male mice but the colon length of fDKO-WT mice was significantly shorter than that of fDWT-WT mice (F = 0.975, df = 14, *p* = 0.046 < 0.05) (Fig. 6F). Hematoxylin and eosin (H.E) staining of the colonic tissue sections showed that DSS-treated colons had great histological damage (cellular infiltration, goblet cell depletion, and damage to the crypt architecture), but the difference between KO-WT and WT-WT mice was unclear. The crypt depth of fDKO-WT mice was lower than that of fDWT-WT mice, but the difference was not significant (Fig. 6G). Figures 6H–K shows that the expression of IL-1β (*p* = 0.028 < 0.05) and IL-10 (F = 0.029, df = 14, *p* = 0.041 < 0.05) in the colon in the fDKO-WT group was significantly higher than that in the fDWT-WT group. Thus, LF-deficient feeding of mice during lactation led to more severe DSS-induced weight loss (female), an increased DAI score (male and female), decreased survival (male mice), and colon shortening (female mice).

Ulcerative colitis is typically associated with dysbiosis of the gut microbiota. Alpha-diversity analysis showed that the Shannon index of the fDKO-WT group was significantly lower than that of the fDWT-WT group. The Chao index at the OTU level of fDKO-WT mice was significantly lower than that of fDWT-WT mice (Fig. 6L). These results suggest that alpha diversity was lower in LF-deficient female mice compared to those with normal milk; no difference was observed in male mice after acute DSS injury. PCoA revealed significant separation between the mDKO-WT and mDWT-WT groups at the OTU level, and the microflora of female LF-deficient DSS mice (fDKO-WT) differed significantly from that of the fDWT-WT group (Fig. 6M). At the phylum level, Bacteroidetes*,* Firmicutes*,* Proteobacteria, Campilobacterota, Desulfobacterota, Actinobacteriota, and Deferribacterota accounted for the majority of the bacteria (Fig. 6N). The relative abundance of Firmicutes was significantly higher in the mDKO-WT group, and of Proteobacteria was significantly higher in the mDWT-WT group. In the fDKO-WT group, the relative abundance of Campilobacterota was significantly higher, whereas that of Desulfobacterota and Cyanobacteria was significantly lower (Fig. 6O). At the genus level, the relative abundance of *Lachnospiraceae_NK4A136 group*, R*uminococcus_torques_group*, *Odoribacter*, *Romboutsia*, *Marvinbryantia*, and *Family_XIII_AD3011_group* was higher in the mDKO-WT group than in the mDWT-WT group (Fig. 6P). As shown in Additional file: Fig. S2A, the abundance of *Ruminococcus_torques _group*, *Odoribacter, Romboutsia*, and *Family_XIII_AD3011_group* was higher in the mDKO-WT group than in the mKO-WT group, indicating that mDKO-WT mice experienced a more substantial increase in these genera. The relative abundances of *Leucobacter* and *Parvibacter* were lower in the mDKO-WT group than in the mDWT-WT group (Fig. 6P). In female mice, the relative abundances of pathogenic bacteria such as *Escherichia-Shigella*, *Enterococcus*, *Clostridium_sensu_stricto_1*, *Erysipelatoclostridium*, *Colidextribacter*, and *Akkermansia* were higher in the fDKO-WT group than in the fDWT-WT group (Fig. 6P). The relative abundances of beneficial genera, such as *Alloprevotella*, *Desulfovibrio*, and many unclassified bacteria on genus level were lower in the fDKO-WT group than in the fDWT-WT group (Fig. 6P). Similarly, the abundance of *Escherichia-Shigella*, *Enterococcus*, *Erysipelatoclostridium*, *Colidextribacter* and *Akkermansia* were increased and *Desulfovibrio* was decreased in the DSS model (Additional file: Fig. S2B). fDKO-WT mice showed a more significant increase in *Escherichia-Shigella*, *Enterococcus*, *Erysipelatoclostridium*, *Colidextribacter* and *Akkermansia* and a serious decline in *Desulfovibrio*. LF feeding-deficient mice clearly showed more severe microbial disorders after DSS processing. In addition, the effects of DSS enteritis on intestinal microbiome of WT mice at genus level are shown in Additional file: Fig. S2C, D.

## Discussion

LF is an important functional protein in breast milk that has profound effects on the growth and development of infants and young children. Formulas are the main breast milk substitute for infants who cannot breastfeed. However, LF has not been widely added to formulas, commonly leading to LF feeding deficiency during lactation. LF has a protective effect on newborns, particularly premature infants. However, the effects of lactation LF feeding deficiency on adult health remain unclear. Depression is the most common mental health problem in adults and a leading risk factor for physical disability. Here, we explored the influence of LF feeding deficiency during lactation on the incidence of depressive-like behavior in adult mice and related mechanisms. Unexpectedly, we found that lactating LF feeding-deficient mice showed more severe depressive behaviour after induction of CUMS depression in adulthood (Fig. 1).

### LF feeding deficiency during lactation impairs intestinal barrier function and immunomodulatory function

Dysfunction of the microbiota–gut–brain axis is the main pathological basis of depression and may directly influence and cause psychiatric disorders [37]. Our results showed that the expression level of tight junction *Zo-1* was significantly affected by lactoferrin intake during lactation after depression in adulthood, and LF feeding deficiency male mice showed more severe inflammation in the colon after adult CUMS. Based on these observations in the intestines, we further explored the effects of LF deficiency during lactation on intestinal development in mice in different growth periods. The small intestine of 18-day-old LF-deficient mice showed decreased intestinal barrier function and innate immunity. ZO family proteins bind directly to F-actin and barrier-forming claudins are necessary for the assembly of tight junctions [38]. Actin cytoskeletal can also regulate epithelial permeability, and knockdown of myosin IIA decreases epithelial permeability in several intestinal epithelial cell lines [39]. Therefore, the low expression of actin protein in the ko-wt group (18-day-old) may have weakened intestinal barrier function. The DEGs related to immunity were mainly enriched in the NOD-like receptor signaling pathway, which plays a key role in pathogen identification and innate immune response (Additional file: Fig. S3). Paneth cells in the crypts of the small intestine secrete microbicidal α-defensins as components of the enteric innate immunity. Moreover, dysregulation of α-defensins has been observed under pathogenic conditions, such as in IBD [40]. The lack of LF during lactation reduces the secretion of α-defensin in the small intestine of suckling mice, which may reduce innate immunity and increase the incidence of inflammatory intestinal disease. When the mice reached adulthood, the difference between the KO-WT and WT-WT groups (mKO-WT vs. mWT-WT, fKO-WT vs. fWT-WT) in the intestinal physiological structure of healthy mice was decreased. However, after DSS enteritis treatment, KO-WT mice showed more serious damage and inflammation, particularly female mice. Thus, loss of LF feeding during lactation hinders the development of intestinal physiological structure, barrier function, and innate immune function in sucking mice, and the gap in intestinal physiological structure gradually narrows with increasing age but a functional gap remains, particularly during negative stress. Therefore, LF feeding deficiency during lactation impairs intestinal barrier and immunomodulatory functions, causing serious damage to the intestinal barrier and inducing systemic inflammation, under the stimulation of depression modelling, which in turn leads to neuroinflammation and depressive-like behavior.

### Effect of LF feeding deficiency during lactation on gut microbiota from infancy to adulthood

The gut microbiota plays a prominent role in gut-brain interactions. The results of gut microbiota are summarized in Additional file: Table. S3. LF feeding deficiency during lactation affects the composition of intestinal microorganisms in mice, and this effect continues into adulthood. The overall microbial composition of 18-day-old ko-wt mice deviated from that of the control group. This separation disappeared in 9-week-old healthy mice, possibly because they had the same dietary conditions. However, this separation reappeared under stimulation of depression and DSS. The effects of LF deficiency during lactation on gut microbes are persistent. Alpha diversity is often used as a proxy for community stability and functioning. The effect of depression on intestinal microbial alpha-diversity is inconclusive [14]. In our study, the microbiota of LF feeding-deficient mice showed lower alpha diversity after CUMS. Similarly, LF feeding-deficient mice showed lower alpha diversity under the influence of DSS enteritis. Alpha diversity was increased in 18-day-old ko-wt mice, possibly because of the antibacterial activity of LF. In contrast, another study showed that LF intervention in early life increased the diversity of the caecal microbiota [20].

LF had no significant influence on the abundance of phylum Firmicutes and Bacteroidota in 18-day-old mice and CUMS model mice. Similar to the results of existing research [22], DSS treatment reduces the level of Firmicutes and increases the level of Bacteroidetes (Additional file: Fig. S4). It is worth noting that Desulfobacterota is enriched in female ko-wt mice in the depression model, but enriched in female wt-wt mice in the DSS model (Additional file: Table. S3). Study found that Desulfobacterota was increased in the depression group [41], the LF feeding deficiency during lactation aggravated the increase of Desulfobacterota caused by depression. Other study showed that Desulfobacterota was increased in the DSS group [42]. However, our study found that Desulfobacterota was decreased in the DSS group, and this phenomenon occurs only in KO-WT mice (mKO-WT vs mDKO-WT, fKO-WT vs fDKO-WT) (Additional file: Fig. S4). The effect of LF deficiency during lactation at the genus level of intestinal microorganisms was complex and diverse. In the intestine of 18-day-old suckling mice, many microorganisms enriched in the ko-wt group were related to inflammation. For example, many studies have reported that *Oscillibacter* is increased in depression [43] and is positively correlated with obesity and increased permeability of the mouse colon [44]. Similarly, the increasing relative abundance of clusters in the ko-wt group, including *Bilophila*, *Colidextribacter*, *Harryflintia* [44], *Odoribacter* [45], *Lachnospiraceae_UCG-006* [46], and *Ruminococcus* [47], has also been reported as positively associated with inflammation due to progressive obesity or type 2 diabetes mellitus. In our study, *Blautia*, *Desulfovibrio*, *Parabacteroides*, *Romboutsia*, *Eubacterium_brachy_group*, and *Turicibacter* were enriched in wt-wt group. *Blautia* may function as a probiotic in the host and has gained attention because of its ability to alleviate inflammatory diseases and metabolic diseases, as well as because of its antibacterial activity against specific microorganisms [48]. However, we found that *Blautia* was positively related to depression. *Parabacteroides* and *Turicibacter* resulted in increased formation of the faecal SCFAs propionate and butyrate [49]. In our RNA-seq results, LF feeding deficiency during lactation decreased the expression of α-defensin in the small intestine of 18-day-old sucking mice (Fig. 3I), which may contribute to host-microbe dysbiosis and enhance inflammatory responsiveness associated with the pathogenesis of IBD [50]. Therefore, the disorder of intestinal microorganisms in 18-day-old ko-wt mice may be directly related to the antibacterial effect of LF. Additionally, it may be indirectly related to the effect of LF on the development and gene expression of the intestine.

When the mice reached adulthood, the abundance of beneficial bacteria in LF-feeding deficient mice was lower than in LF-fed mice. Among the intestinal microorganisms of 9-week-old healthy mice, the abundance of *Kurthia*, *Enterorhabdus*, *Candidatus_Saccharimonas*, *Monoglobus*, *ASF356*, and *Eubacterium_xylanophilum_group* was lower in the mKO-WT group and decreased in the DSS model compared to in healthy mice (Additional file: Fig. S2A). A previous study showed that the abundance of E*nterorhabdus spp.* decreased after chronic DSS induction [51]. According to previous reports, *ASF356* exerts probiotic effects [52]. *Candidatus_Saccharimonas* can suppress the production of TNF-α in macrophages, suggesting its potential for immune suppression [53]. *Eubacterium_nodatum_group* enriched in fWT-WT is SCFAs-producing bacteria [54]. These results suggest that the adverse effect of LF feeding deficiency during lactation on the composition of intestinal microorganisms continued into adulthood. This effect was also observed in the DSS model. Among intestinal microorganisms in the DSS model, more harmful bacteria were enriched in LF-deficient mice (Fig. 6P). *Ruminococcus_torques_group* and *Family_XIII_AD3011_group* were enriched in the mDKO-WT group; these genera are significantly positively corelated with inflammation [24, 55]. *Escherichia-Shigella, Enterococcus, Clostridium_sensu_stricto_1*, *Erysipelatoclostridium*, *Colidextribacter* and *Akkermansia* were increased in the fDKO-WT group compared to in the fDWT-WT group. *Escherichia-Shigella* and *Enterococcus* are potential pathogens [56, 57], and studies found that *Clostridium_sensu_stricto_1*, *Erysipelatoclostridium* and *Colidextribacter* were increased in high-fat diet-fed mice [44, 45, 58]. Similar to our results, Xu et al. found that the relative abundance of *Akkermansia* was markedly increased after chronic DSS induction [51]. Considering the close relationship between the intestinal microbial composition and depression, LF deficiency during lactation is likely to affect intestinal microorganisms in the CUMS model. In the CUMS model, *Eubacterium_xylanophilum_group* was enriched in the ko-wtM group and was positively correlated with FST (Fig. 2L), which was positively correlated with the severity of depression. Study found that *Eubacterium_xylanophilum_group* was the characteristic genera of valproic acid-induced rat autism model [59]. To the best of our knowledge, our results are the first to report that *Eubacterium_xylanophilum_group* might be associated with depression. We also found that *Alistipes* was positively correlated with the OFT, indicating that *Alistipes* is negatively correlated with the severity of depressive symptoms. The abundance of *Alistipes* is decreased in patients with IBD, which is characterised by overexpression of indoleamine 2,3-dioxygenase (IDO) in the colon [60]. IDO can reduce 5-hydroxytryptamine (5-HT) levels by promoting the metabolism of tryptophan, a precursor of 5-HT synthesis, leading to depression. In our study, the abundance of *Lactobacillus* was decreased in the ko-wtM group but increased in the ko-wtF group (Fig. 2K). Studies of the role of *Lactobacillus* in depression have reported conflicting results. Mice with chronic restraint stress-induced depressive-like behavior showed a significant increase in *Lactobacillus* [61], whereas a low abundance of *Lactobacillus* was found in people with depression [62]. In addition, this difference may be sex-dependent, and *Lactobacillus* was shown to be inversely associated with depression scores among males but was positively associated with anxiety scores among females [63], which is similar to our results. *Bifidobacterium* was enriched in the ko-wtF group, and is present at high levels in major depressive disorder/depression groups in many studies [14]. Interestingly, specific strains of *Bifidobacterium* are commonly considered as anti-inflammatory and used as probiotics. However, a higher abundance of *Bifidobacterium* has been associated with IBD, indicating that specific strains have inflammatory potential [64]. However, we found that *Bifidobacterium* was negatively corelated with depressive-like behavior (Fig. 2L), based on the negative correlation between *Bifidobacterium* and depressive-like behavior in male mice (Additional file: Fig. S5), and the correlation was greater than that in female mice, thus affecting the overall correlation. *Bifidobacterium* was also enriched in the 18-day-old ko-wt (Fig. 3O) group and 9-week mKO-WT (Fig. 5M) group, indicating that colonization of the neonatal intestine by *Bifidobacterium* can be reduced if milk is concurrently supplemented with bovine LF [65]. Therefore, a lack of LF during lactation may be beneficial for colonization of *Bifidobacterium*. Many studies revealed higher levels of *Desulfovibrio* in patients with major depressive disorder than in controls [14, 62]. Similarly, *Desulfovibrio* was enriched in ko-wtF group (Fig. 2K) and was positively related to the severity of depressive symptoms in our study (Fig. 2L).

The lack of LF during lactation increased the pro-inflammatory microflora in the intestinal microflora of 18-day-old mice and decreases the number of beneficial bacteria in adult mice, which is disadvantageous for establishing a healthy intestinal ecosystem. LF feeding deficiency during lactation may decreased the stability of intestinal microorganisms, rendering them more vulnerable to dysregulation during adverse stimuli. However, further investigation is needed to understand how the impact of lactoferrin on microbial composition translates into host health. Future studies should consider conducting metabolomic analysis to shed more light on this aspect.

### LF feeding deficiency during lactation inhibits hippocampal development from infancy to adulthood

The hippocampus is a key brain region involved in cognitive processes, mood regulation, and the pathophysiology of depression [12]. In our study, the ko-wtF group exhibited more severe hippocampal inflammation (Fig. 2E) and lower BDNF levels (Fig. 1I, 2F). In the hippocampus of 18-day-old mice, genes involved in innate immunity were downregulated in the ko-wt group (Fig. 4F). Family-wide loss-of-function analysis showed that *Gbp10* conferred cell-autonomous immunity to listerial or mycobacterial infection within macrophages and gene-deficient animals [27]. Another study revealed that *stx11* knockdown reduced phagocytosis of *Escherichia coli* in interferon-γ-activated macrophages [28]. The innate immune response can eliminate most invading pathogenic microorganisms and participate in immune self-stabilization of the body. Low expression of innate immune related genes is unfavorable for pathogen clearance in time, thus aggravating infection. Furthermore, activation of innate immunity is indispensable for inducing adaptive immune responses. Downregulation of innate immune function may affect the intensity and type of adaptive immunity. In our study, the genes involved in adaptive immunity were upregulated in the hippocampus of LF-deficient mice (Fig. 4F). *Ccl21a* promotes the establishment of self-tolerance in T cells in the thymic medulla [29]. *CD28* is the second signal required for T-cell activation during the immune response [31]. *CD247* is part of the T-cell antigen receptor complex, which plays a key role in receptor expression and signaling leading to optimal effector T-cell functions [30]. *IL-25* can induce and enhance Th2 type immune response [32]. Some genes participate not only in the innate immunity of the mucous membrane but also in acquired immunity. *Pigr* mainly functions to cooperate with IgA transport, which plays an important role in mucosal immunity [33]. *CCL28* is an anchoring point that bridge innate and adaptive immunities [36]. The *Ltf* gene encodes LF, which has a defensive role in the body as its levels were found to be profoundly elevated under pathological conditions such as neurodegeneration and inflammatory disease [66]. *Lcn2* can control neuroinflammation by modulating cytokine production and exerts neuroprotective functions in the brain by suppressing the secretion of pro-inflammatory cytokines [34]. *S100a9* encodes a pro-inflammatory protein (calgranulin) that has been implicated in multiple diseases. A previous study has shown that *S100a9* prevents hyperinflammatory responses without impairing pathogen defence [35]. The *Ltf*, *Lcn2*, and *S100a9* genes play important roles in controlling the balance of the immune response, and low expression of these genes may induce immune response disorders. Although our results showed that the disorder of hippocampal immunomodulatory function caused by LF feeding deficiency in 18-day-old mice disappeared in adult mice (Fig. 4I), another study showed that early life inflammation causes depressive symptoms in adolescence [67], suggesting that disorder of hippocampal immunomodulatory function likely led to neuroinflammation in 18-day-old ko-wt mice, which may have been related to depressive-like behavior in adult mice.

Mice fed an LF-deficient diet during lactation may be at risk of neurodevelopmental delay. *Prdm12* regulates the proliferation and differentiation of neural stem cells. Overproduction of *prdm12* impaired cell proliferation and increased the G1 population [68], and high expression of *Prdm12* in the ko-wt group (18-day-old mice (Fig. 4G) may inhibit neurogenesis. *Pcdha8* is a member of the protocadherin cluster, which regulates the ability of neurons to migrate and of dendrites to avoid each other. Many brain diseases, such as autism spectrum disorders and depression, are caused by abnormal neuronal migration and connections [69]. Low *Pcdha8* expression increases the risk of depression [69], but the influence of high *Pcdha8* expression on depression is unclear. In our study, the expression of *Pcdha8* was higher in the ko-wt group (18-day-old mice (Fig. 4G)). The *Cdh3* gene encodes cadherin, a cell adhesion molecule that is important in a variety of morphogenetic events during neural development including cell migration, segmentation of the neural tube, neurite outgrowth, axon targeting, and synapse formation [70]. A previous study showed that the overexpression of *Wisp2/Ccn5* potently enhanced neurite outgrowth. The low expression of *Cdh3* and *Ccn5* in 18-day-old ko-wt mice (Fig. 4G) may negatively affect neural development. We found that *BDNF* expression was significantly lower in the 18-day-old ko-wt mice (Fig. 4G); similarly, the ko-wtM and ko-wtF mice showed lower BDNF serum levels after CUMS (Fig. 1I). LF has been suggested to promote early neurodevelopment and cognition in postnatal piglets by upregulating the *BDNF* signalling pathway [71]. Therefore, a lack of LF during lactation reduces the expression of *BDNF*, which may increase the risk of depressive-like behavior in adult mice. In the hippocampi of 18-day old mice, the expression of *Folr1* was lower in the ko-wt group (Fig. 4G). *Folr1* is a membrane protein that mediates folic acid uptake by cells. Recent studies have shown that mouse *Folr1* is essential for neural tube closure and can induce central nervous system axon regeneration mediated by folate [72].

In our study, the expression of genes related to neuronal signal transduction in the 18-day-old ko-wt mice significantly decreased (Fig. 4H), but did not differ from that in adult mice (Fig. 4K). *Gabra6* is a GABA receptor. GABA is a neurotransmitter involved in the mechanism of anxiety and depression. A rat experiment suggested that depressive behaviour caused by maternal-foetal separation is related to decreased expression of *Gabra6* [73], and low GABA transmission has been reported in victims of suicide [74]. *TRPV4* is a Ca2^+^ nonselective cation channel involved in modulating membrane potentials in neurons. A previous study showed that downregulation of hippocampal *TRPV4* affects depression-like behavior in mice by regulating neurogenesis [75]. *Kcne2* and *Kcnj13* regulate the return of the membrane potential to resting potential, and loss of *Kcne2* can increase excitability in neurones [76]. Decreased expression of *Kcne2* and *Kcnj13* may lead to continuous excitation of neurons, resulting in neurotoxicity. In summary, LF feeding deficiency during lactation decreased the expression of *Gabra6*, *TRPV4*, *Kcne2*, and *Kcnj13* in 18-day-old mice, which may increase the depressive-like behavior rate of in mice; however, as the mice aged, this difference disappeared. The choroid plexus is the site of cerebrospinal fluid (CSF) production and of the blood–CSF barrier, and claudin2 is present in the epithelial tight junctions of the choroid plexus forming the blood–CSF barrier [77]. Our results showed that *Cldn2* expression was decreased in 18-day old ko-wt mice (Additional file: Fig. S6), which may have led to increase in permeability of the blood–CSF barrier and thus more severe neuroinflammation and an increased risk of depressive-like behavior.

### Sex differences

We evaluated mice of both sexes and observed differences in some results between male and female mice. Epidemiological studies have shown that women have much higher rates of major depression and higher levels of inflammatory markers than men [78]. Similarly, female mice showed a more severely depressed phenotype in our study (Fig. 1I-J). Furthermore, female mice had lower intestinal tight junction expression and higher level of inflammatory cytokines (Fig. 2B, Fig. 1M-N), which induced more severe hippocampal inflammation compared to in male mice in our CUMS model (Fig. 2E). LF-feeding deficient male mice showed more severe intestinal inflammatory damage after CUMS (Fig. 2). In contrast, the LF-feeding deficient female mice showed mild intestinal inflammatory damage after CUMS. This difference may be related to the anti-inflammatory effect of oestrogen, study found that oestrogen decreased the production of macrophage migration inhibitory factor, which affected the susceptibility to inflammation in the colon by reducing the TNF-α and IL-1β production in colitis [23, 79]. However, why did the anti-inflammatory effects of oestrogen manifest only in the ko-wtF and not in wt-wtF mice? We speculated that the LF feeding deficiency during lactation may influenced the signal transduction of estrogen. In 18-day-old suckling mice, the genes encoding Fkbp5 and Hsp70 in the oestrogen signalling pathway were up regulated in the ko-wt group (Additional file: Fig. S7). As receptor associated proteins, Fkbp5 and Hsp70 affect oestrogen receptor nucleocytoplasmic shuttling [80]. Oestrogen receptors are abundantly expressed in the intestine and cells of the immune system [81], and binding of these receptors to oestrogen induces gene regulation. This phenomenon did not occur in the DSS enteritis model, possibly because of the different pathogeneses of chronic enteritis caused by depression and acute enteritis established by DSS. Further studies are needed to investigate the underlying reasons for this result. In our DSS model, in agreement with the results of previous studies [79], male mice showed more severe weight loss and higher mortality than female mice, which was also related to the anti-inflammatory function of oestrogen. Sex-specific relationships between gastrointestinal microbiota and depressive-like behavior were also observed in this study. The abundance of *Lactobacillus* and *Bifidobacterium* was negatively related to depressive-like behavior among male mice and positively related to depressive-like behavior among female mice. Currently, the relationship between *Lactobacillus* and *Bifidobacterium* and depression in individuals of different sex is still inconclusive, which is also an important reason to prevent these two bacteria from being widely used in the clinical treatment of depression [63].

## Conclusions

Our study demonstrated for the first time that LF feeding deficiency during lactation increases the risk of depressive-like behavior in adults. During negative stress, LF deficiency causes more serious inflammatory responses, thus increasing systemic inflammation and neuroinflammation, leading to depressive-like behavior. Lack of LF during lactation disrupts the homeostasis of intestinal microorganisms in mice from infancy to adulthood and causes more serious flora disorders after negative stress stimulation. Additionally, lack of LF during lactation leads to neuroimmune dysfunction and neurodevelopmental retardation in the hippocampus, which in turn leads to increased susceptibility to depressive-like behavior. In short, LF feeding deficiency during lactation increases the risk of depressive-like behavior in adult mice by inducing microbiota–gut–brain axis disorders.

## Methods

### Animal

The heterozygous *Ltf* gene knockout mice were customized from Biocytogen Co., Ltd. (Beijing, China). Heterozygous breeding produced *Ltf* gene knockout (KO) and wild-type littermate (WT) C57BL/6N mice, which were used for homozygous breeding to produce a larger number of age-matched WT and KO mice for the current experiments. Before the experiment, KO and WT homozygous breeding produced mice at the same time. On the second day after birth, all litters born to KO dams were replaced with pups of the same age born to WT dams (KO-WT group). Control litters suckling WT dams were also standardized to pups on the second day after birth (WT-WT group). Offspring were weaned and seperated from mothers on d21, males and females separated into cages of 4 mice each cage on 4 weeks. The mice in KO-WT group drank LF-free milk during sucking time, and these two group mice would be used in subsequent experiments.

Mice were housed in a controlled environment (12:12 h light: dark cycle; temperature: 22 ± 2 °C; humidity: 60%; 15 − 20 fresh air changes per hour) with free access to food (fed with sustaining fodder) and water. Cage cleaning was performed once per week. Each cage size is 25 cm × 15 cm with four mice per cage. All mice were treated in accordance with the guidelines of the Institutional Animal Care and Use Committee (SYXK 2020–0052). All experiments were approved by the Animal Experimentation Committee of the China Agricultural University (Beijing, China).

### Procedure of CUMS

16 male 9-week-old mice (8 from WT-WT group and 8 from KO-WT group) and 16 female 9-week-old mice (8 from WT-WT group and 8 from KO-WT group), were divided into 4 groups: wt-wtM, ko-wtM, wt-wtF, ko-wtF (Figs. 1 and 2). The CUMS procedure was a variation of methods described by Agnieszka [82]. The list of stressors, their details, and the day they were applied was given in Additional file: Table. S4.

### Behavioral evaluations

Open field test (OFT).

The test consisted of one 5 min trial in a white opaque 50 cm × 50 cm × 50 cm arena. The center zone was defined as a 25 cm × 25 cm central square. To begin each test, a mouse was introduced to the center of the square and its behavior was captured on video (Samsung, South Korea) over the course of 5 min, the area was cleaned with 50% ethanol and allowed to dry completely between each test. The distance that each mouse walked in either the peripheral or central regions was quantified using KEmaze software.

### Sucrose preference test (SPT)

The sucrose preference test was performed according to a previous report [83] with minor modifications. Before the test, mice were acclimatized to sucrose solution. At first, two bottles of 1% sucrose solution (w/v) were placed in each cage for 24 h to avoid sucrose neophobia. After adaptation, mice were deprived of liquid and food for 24 h and then given the sucrose preference test. Each mouse had free access to 2 bottles for 24 h: one bottle with 1% sucrose solution (w/v) and another bottle with tap water. To avoid position effects, the positions of the 2 bottles were reversed at intervals of 12 h. Sucrose consumption and water consumption were measured by comparing the weights of the bottles before and after the test. The sucrose preference was calculated as sucrose preference (%) = (sucrose solution intake)/ (sucrose solution intake + tap water intake) *100%.

### Forced swimming test (FST)

Each mouse was placed in an open cylindrical container (diameter 10 cm, height 30 cm) with 23 ± 1℃ water to a depth of 20 cm and allowed to swim for 6 min. Immobility times were measured during the last 4 min of the test. During the test, the behavior of each mouse was recorded using a video camera (Samsung, South Korea), and immobility time was measured using SuperFst software (KEWBIO Co., Ltd. Nanjing, China).

### Tail suspension test (TST)

Mice were habituated to the testing room for 30 min before the experiments. Animals were attached by their tails to a shelf with medical adhesive tape (placed approximately 1 cm from the distal end of the tail). The last 4 min was recorded for immobility time during a 6-min test. During the test, the behavior of each mouse was recorded using a video camera (Samsung, South Korea), and immobility time was measured using SuperTst software (KEWBIO Co., Ltd. Nanjing, China).

### Dextran Sodium Sulfate (DSS)-induced colitis

Colitis was induced by the administration of 2.5% w/v DSS (MW = 36,000–50,000 kDa; MP Biomedicals) in the drinking water for 7 days [12]. The 8-week-old female mice (fDWT-WT, fDKO-WT) and male mice (mDWT-WT, mDKO-WT) were weighed daily and the disease activity index (DAI) score can be assessed (Fig. 6). The DAI is the combined score of weight loss compared to initial weight, stool consistency and bleeding [84].

### Sample collection and measurement

After 4 weeks of CUMS, all animals were sacrificed under anesthesia with 10% chloral hydrate and the colon tissue, hippocampus, serum and cecal content of all animals were collected and stored at − 80 °C for the following experiments. Blood was collected from the retro-orbital sinus under 10% chloral hydrate general anesthesia. Serum was obtained from the whole blood by centrifugation at 5000 rpm for 20 min. Collected colon samples were fixed in 4% paraformaldehyde and embedded in paraffin. Sections of the colon were stained with H.E. The inflammation score (0: normal cell pattern, 1: scattered inflammatory cells in the lamina propria, 2: increased number of inflammatory cells in the lamina propria, 3: confluence of inflammatory cells extending into the submucosa, 4: transmural extension of the infiltrating inflammatory cells) [85].

18-day-old mice (wt-wt, ko-wt) were sacrificed under anesthesia with isoflurane without fasting. The body weight, whole brain, small intestinal and colon were weighted, length of small intestinal and colon were measured. The small intestinal, colon, hippocampus and cecal content were collected and immediately snapfrozen in liquid nitrogen. Collected colon and hippocampus samples were fixed in 4% paraformaldehyde and embedded in paraffin. Sections of the colon were stained with H.E and alcian blue (AB) staining by using commercial kits (Beijing Solarbio Technology Co., Ltd. Beijing, China) according to the manufacturer's instructions. Paneth cells staining by using Lendrum's fluorescent Peach Red staining Kit (Xi’an Qiyue biology, Xian, China) Sections of the hippocampus were stained with H.E. (Figs. 3 and 4A-H).

9 weeks female mice (fWT-WT, fKO-WT) and male mice (mWT-WT, mKO-WT) (Figs. 4I-K and 5) were sacrificed under anesthesia with 10% chloral hydrate. The small intestine and colon were removed, and total length and weight were measured. Intestinal density is indices of intestinal mucosal growth. Intestinal density = weight of small intestine or colon/length of small intestine or colon. The small intestinal, half of colon, hippocampus and cecal content were collected and immediately snap-frozen in liquid nitrogen. The other half of colon was fixed in 4% buffered formaldehyde for the histological observation. The activities of lactase and maltase in each section of the small intestine were measured using commercial kits (Nanjing Jiancheng Institute of Bioengineering, Jiangsu, China) according to manufacturer’s instructions. Sections of the intestinal were stained with H.E.

The DSS induced colitis mice (mDWT-WT, mDKO-WT, fDWT-WT, fDKO-WT) (Fig. 6) were sacrificed on day 7 and colon length was measured. Half of colon and cecal content of all animals were collected and stored at − 80 °C for following experiments. The other half of colon was fixed in 4% buffered formaldehyde for the histological observation. Cytokine levels in colonic tissues were detected by enzyme-linked immunosorbent assay (ELISA) kit (Cloud-Clone, Wuhan, China) according to the manufacturer's instructions.

### RNA sequencing

The small intestinal and hippocampus of 18-day-old mice (wt-wt, ko-wt) were collected and the total RNA was extracted by using TRIZOL reagent (Invitrogen, USA). The concentration and purity of the RNA were detected by Nanodrop2000, RNA integrity was detected by agarose gel electrophoresis, the RIN value was measured by Agilent2100. The construction of a single database requires that the total amount of RNA ≥ 1 μg, concentration ≥ 35 ng/μl, OD260/280 = 1.8 ~ 2.2, OD260/230 ≥ 2.0.RIN ≥ 6.5, 28S:18S ≥ 1.0, Libraries were constructed using the Illumina TruseqTM RNA sample prep Kit (Illumina, San Diego, CA, USA) according to the manufacturer’s instructions.

Sequencing of the libraries was performed on an Illumina HiSeq2000 instrument by Shanghai Majorbio Biopharm Biotechnology (Shanghai, China), and individually assessed for quality using FastQC. To identify DEGs between two different samples, the expression level of each transcript was calculated according to the transcripts per million reads (TPM) method. RSEM (http://deweylab.biostat.wisc.edu/rsem/) was used to quantify gene abundances. Differential expression analysis was performed using the edgeR with Q value ≤ 0.05. Statistical significance was assessed using a negative binomial Wald test, then corrected for multiple hypothesis testing with the Benjamini–Hochberg method.

### Quantitative real-time PCR

Total RNA was extracted from the tissue using an RNA extraction kit (Magen, Guangzhou, China) according to the manufacturer’s instructions. The purity and concentration of RNA were determined using a NanoDrop spectrophotometer (Thermo Fisher, Waltham, MA). One microgram of total RNA was reverse transcribed into cDNA using a HiFiScript cDNA synthesis kit (NUOWEIZAN, Nanjing, China). RT-PCR was performed using Rotor-Gene Q (Qiagen, Hilden, Germany) with an Taq Pro Universal SYBR qPCR Master Mix Kit (NUOWEIZAN, Nanjing, China). RT-PCR amplification conditions included denaturation at 95 °C for 10 s, annealing at 55 °C for 30 s, and extension for 32 s at 72 °C. Geometric mean glyceraldehyde-3-phosphate dehydrogenase (*Gapdh*) was used for normalization. All primer pairs were synthesized by Synbio Technologies (Jiangsu, China), the sequence was showed in Table 1.

**Table 1:** Sequences of primers for real-time PCR

Gene name	F-primer (5’-3’)	R-primer (5’-3’)
*Gapdh*	TCTCCTGCGACTTCAACA	TGTAGCCGTATT CATTGTCA
*Zo-1*	GCATCATTCGCCTTCATAC	GACACAACCTCATCCTCAT
*Occludin*	GGCTGCTGCTGATGAATA	ATCCTCTTGATGTGCGATAA
*TLR4*	CACTGTTCTTCTCCTGCCTGA	GGAATGTCATCAGGGACTTTGC
*LBP*	GTCGTGGGCAGTACGAGTTT	CCTTCCATTTGCCTCGGACA
*MyD88*	CGCATGGTGGTGGTTGTTTC	AGTCGCTTCTGTTGGACACC
*CD14*	ACAATTCACTGCGGGATGCT	AGCTCATCTGGGCTAGGGTT
*JNK*	CCACCAAAGATCCCGGACAA	GGCTGCCCTCTTATGACTCC
*NFκB*	CCCTACGGAACTGGGCAAAT	GCAAATTTTGACCTGTGGGT
*IκBα*	CCTGACCTGGTTTCGCTCTT	CTGTATCCGGGTACTTGGGC
*P38MAPK*	TTCTACCGGCAGGAGCTGAA	ATCAAAAGCAGCACACACCG
*IL-1β*	CTTCAGGCAGGCAGTATC	CAGCAGGTTATCATCATCATC
*TNF-α*	ACTGAACTTCGGGGTGATCG	CCACTTGGTGGTTTGTGAGTG
*Bdnf*	TAAACGTCCACGGACAAGGC	AGTGTCAGCCAGTGATGTCG
*Grb2*	AACATCCGTGTCCAGGAACC	AAGTCTCCTCTGCGAAAGCC
*CaMK1*	TGATCCTGGCAGAGGACAAGA	GCTACAATGTTGGGGTGCTTG
*Creb*	AACCAGCAGAGTGGAGATGC	GATGTTGCATGAGCTGCTGG
*Lrg1*	TGTCCAGCCTCAAGGAATGC	TTCCACCGACAGATGGACAG
*Gbp4*	TGAACCAGGAAGCCATAGAGA	GGGAAACCTTTGGCTGGTAGG
*Stx11*	ATGACTTTGACGCTCCTCGG	GCGCCTCACGTCTATCAGAA
*Pigr*	GACTCTCGCTGGAGAACCAC	ACGGATAGTGGCAGGAAACG
*Ltf*	AGCTGAAGTCTACGGGACCA	CTCAGGCCTTGGAGTTGGTT
*Lcn2*	ACAACCAGTTCGCCATGGTAT	AAGCGGGTGAAACGTTCCTT
*S100a9*	ACCACCATCATCGACACCTTC	AAAGGTTGCCAACTGTGCTTC
*Ccl28*	CTCACACTCATGGCTGTGGCT	AGTACGATTGTGCGGGCTGA
*IL-25*	GACCTGTACCACGCTCGATG	AGAAGACCGTCTGGTTGTGG
*Ccn5*	TGTGTGACCAGGCAGTGATG	GTGCTCCAGTTTGGACAGGG
*Cdh3*	GCATGCACCATGCAGACAAT	GTGGCATCACCCACTCTCTC
*Prdm12*	TCAAGTGTGCCCGGAATGAG	TCCTGGTCTGGAGGGATCAT
*Pcdha8*	TCAAAGGGTTTTGGGGACCT	GAGCCTTTTGTGTCGCTGGA
*Trpv4*	TGGAACCAGAACTTGGGCAT	GGACCAACGATCCCTACGAA
*Gabra6*	AGGAGTCAGTCCCAGCAAGA	ATGAACCAATCCATGGCGGT
*Kcne2*	CCAGAGTGGATGCCGAGAAC	CTTCGACTTCACCGTGCTCA
*Kcnj13*	TTTGTGGCGAAGATTGCACG	ACACGAACGTTGGTCAGAGG

### 16S rRNA gene sequencing and analysis

Microbial community genomic DNA was extracted from cecal content samples using the E.Z.N.A.® soil DNA Kit (Omega Bio-Tek, Norcross, GA, USA) according to the manufacturer’s instructions. The DNA extract was checked on 1% agarose gel, and DNA concentration and purity were determined with NanoDrop 2000 UV–vis spectrophotometer (Thermo Scientific, Wilmington, USA). The hypervariable region V3-V4 of the bacterial 16S rRNA gene were amplified. The PCR product was extracted from 2% agarose gel and purified using the AxyPrep DNA Gel Extraction Kit (Axygen Biosciences, Union City, CA, USA) according to the manufacturer’s instructions and quantified using Quantus™ Fluorometer (Promega, USA).

Purified amplicons were pooled in equimolar and paired-end sequenced on an Illumina MiSeq PE300 platform/NovaSeq PE250 platform (Illumina, San Diego, USA) according to the standard protocols by Majorbio Bio-Pharm Technology Co. Ltd. (Shanghai, China).

The raw 16S rRNA gene sequencing reads were demultiplexed, quality-filtered by fast version 0.20.0 and merged by FLASH version 1.2.7. Operational taxonomic units (OTUs) with a 97% similarity cutoff were clustered using UPARSE version 7.1, and chimeric sequences were identified and removed. The taxonomy of each OTU representative sequence was analyzed by RDP Classifier version 2.2 against the 16S rRNA database using a confidence threshold of 0.7.

### Statistical analysis

The results are expressed as mean ± SEM. Statistical analyses were performed using IBM SPSS Statistic 23. The data were tested for normality using the Shapiro–Wilk normality test. Data that passed the normality test were analyzed using Students two-tailed t-test and ANOVA (two-way ANOVA and repeated measures two-way ANOVA). While non-parametric Mann–Whitney U tests were performed on samples that did not pass the Shapiro–Wilk normality test (*P* < 0.05). Statistical significance was set at *P* < 0.05.

### Supplementary Information

**Additional file 1:** **Fig. S1.** Paneth cell numbers in the colon of 18-day-old mice and representative images of fluorescence red tartar yellow stained colonic sections. **Fig. S2. **Analysis of species difference at intestinal microbial genus level between DSS enteritis mice and normal mice. **Fig. S3**. KEGG pathway enrichment analysis of immune related differential genes in 18-day-old mice small intestinal. **Fig. S4.** The abundance of phylm Firmicutes, Bacteroidota, Desulfobacterota in 18-day-old mice, 9-week-old mice, DSS model mice and CUMS depression mice. **Fig. S5.** The abundance of Bifidobacterium in CUMS mice. **Fig. S6**. Expression of Cldn2 in the hippocampus RNAseq of 18-day-old mice. **Fig. S7.** Expression of genes encode FKBP5 and HSP70 in 18-day-old small intestinal RNAseq. **Table. S1.** The R value of Fig.2L. **Table. S2.** The P value of Fig.2L. **Table. S3.** The summary of gut microbiota results in our study. **Table. S4.** Chronic unpredictable mild stress schedule.

## Data Availability

All data generated or analyzed during this study are included in this published article, its supplementary information files and publicly available repositories. The raw reads have been deposited at the NCBI Short Read Archive with the project accession numbers: PRJNA1030344 [86] (Effects of lactoferrin deficiency during lactation on intestinal microbial composition in adult depressed mice), PRJNA1030310 [87] ( Effects of lactoferrin deficiency during lactation on intestinal microbial composition in 18-day mice, 9-week health mice and DSS incuced colitis mice), PRJNA1030326 [88] (Effect of lactoferrin deficiency during lactation on small intestinal transcriptome in mice), PRJNA1030504 [89] (Effect of lactoferrin deficiency during lactation on small intestinal transcriptome in mice).

## Abbreviations

5-HT: Hydroxytryptamine
AB: Alcian blue;
ACTH: Adrenocorticotropic hormone
BDNFs: Brain-derived nutritional factors
CORT: Corticosterone
CSF: Cerebrospinal fluid
CUMS: Chronic unpredictable mild stress
DAI: Disease activity index
DEGs: Differentially expressed genes
DG: Dentate gyrus
DSS: Dextran sodium sulfate
ELISA: Enzyme-linked immunosorbent assay
FST: Forced swimming test
GABA: γ-Aminobutyric acid type A
GAPDH: Glyceraldehyde-3-phosphate dehydrogenase
H.E: Haematoxylin and eosin
IBD: Inflammatory bowel disease
IDO: Indoleamine 2,3-dioxygenase
IL: Interleukin
KEGG: Kyoto Encyclopedia of Genes and Genomes
KO: Knockout
LF: Lactoferrin
LPS: Lipopolysaccharide
MIF: Macrophage migration inhibitory factor
NF-κB: Nuclear factor-κB
OFT: Open field test
OTU: Operational taxonomic unit
PCoA: Principal coordinate analysis
RT-PCR: Quantitative real-time PCR
SCFAs: Short-chain fatty acids
SPT: Sucrose preference test
TLR: Toll-like-receptor
TNF-α: Tumour necrosis factor-α
TPM: Transcripts per million reads
TST: Tail suspension test
WHO: World Health Organization
WT: Wild-type
zo-1: Zonula occludens
