# Supplementary material for: Lactoferrin deficiency during lactation increases the risk of depressive-like behavior in adult mice
Source: BMC Biol. 2023 Oct 31;21:242. doi: 10.1186/s12915-023-01748-2 (PMC10617225; doi:10.1186/s12915-023-01748-2)
Supplement: Supplementary file 1 — Additional file 1: Fig. S1. Paneth cell numbers in the colon of 18-day-old mice and representative images of fluorescence red tartar yellow stained colonic sections. Fig. S2. Analysis of species difference at intestinal microbial genus level between DSS enteritis mice and normal mice. Fig. S3. KEGG pathway enrichment analysis of immune related differential genes in 18-day-old mice small intestinal. Fig. S4. The abundance of phylm Firmicutes, Bacteroidota, Desulfobacterota in 18-day-old mice, 9-week-old mice, DSS model mice and CUMS depression mice. Fig. S5. The abundance of Bifidobacterium in CUMS mice. Fig. S6. Expression of Cldn2 in the hippocampus RNAseq of 18-day-old mice. Fig. S7. Expression of genes encode FKBP5 and HSP70 in 18-day-old small intestinal RNAseq. Table. S1. The R value of Fig.2L. Table. S2. The P value of Fig.2L. Table. S3. The summary of gut microbiota results in our study. Table. S4. Chronic unpredictable mild stress schedule. [file 12915_2023_1748_MOESM1_ESM.docx]

Additional file for

**Lactoferrin Deficiency during Lactation Increases the Risk of Depression in Adult Mice**


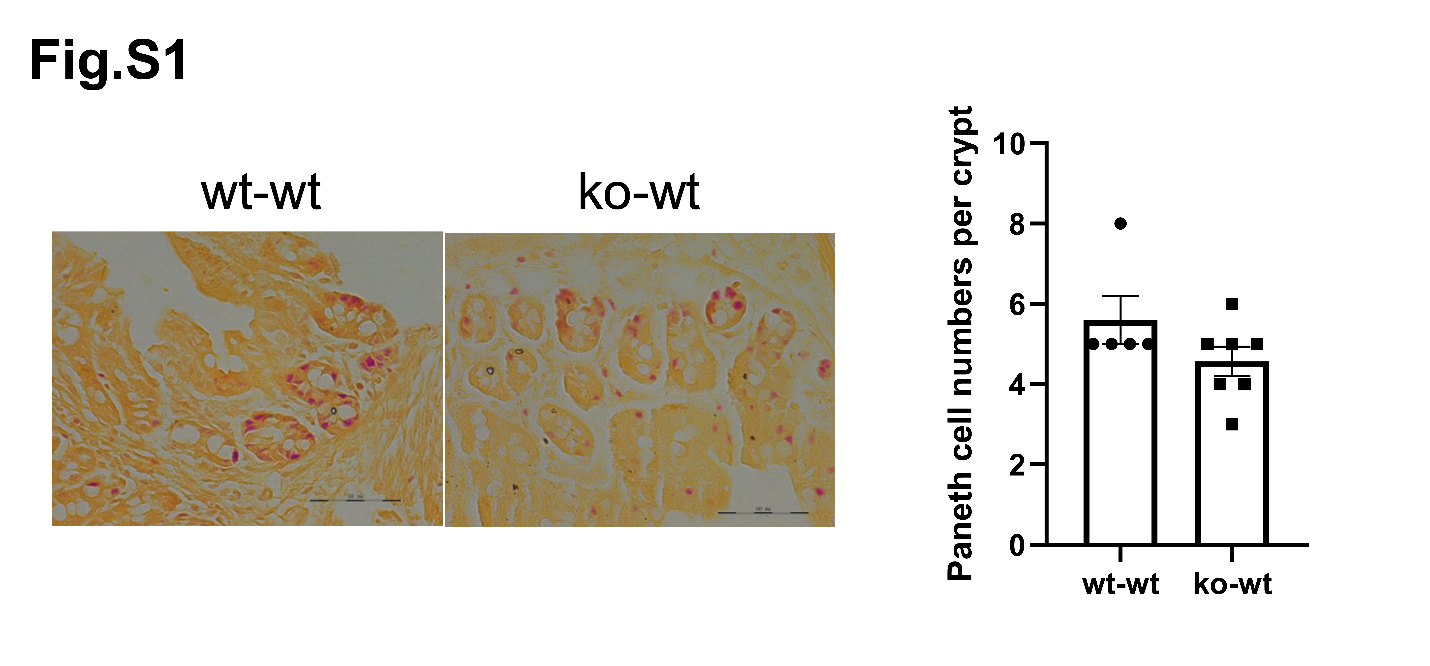
**Fig. S1** Paneth cell numbers in the colon of 18-day-old mice and representative images of fluorescence red tartar yellow stained colonic sections, 400×, scale bar = 50 μm. The red dots in the picture are Paneth cells. n=5-7.

**
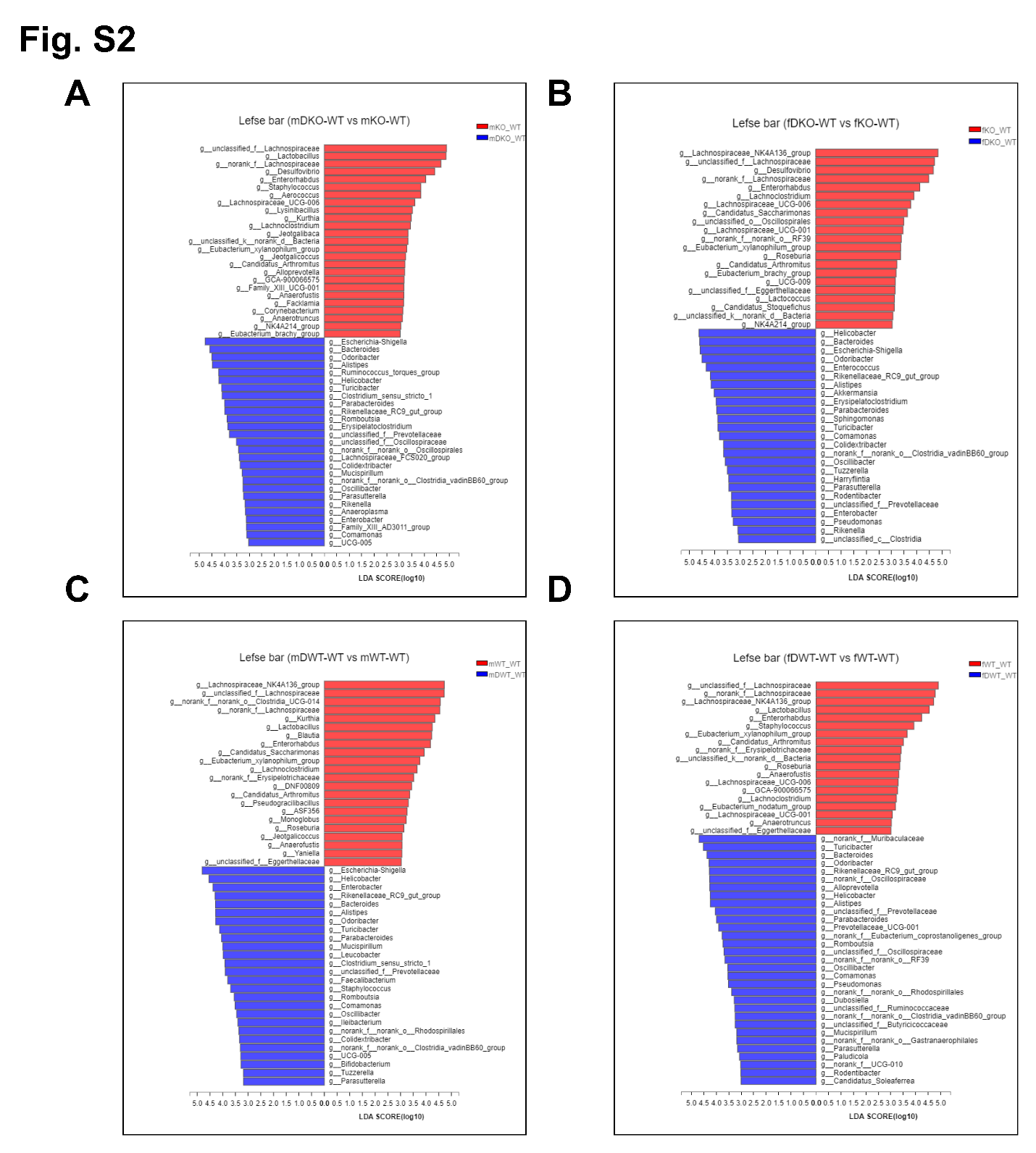
Fig. S2**. Analysis of species difference at intestinal microbial genus level between DSS enteritis mice and normal mice. (A) . Linear discriminant analysis (LDA >3) scores derived from LEfSe analysis at genus level to show the different bacteria between DSS model male mice and health adult male mice that drink LF-free milk during lactation (mDKO-WT vs mKO-WT ). (B). Linear discriminant analysis (LDA >3) scores derived from LEfSe analysis at genus level to show the different bacteria between DSS model female mice and health adult female mice that drink LF-free milk during lactation (fDKO-WT vs fKO-WT). (C). Linear discriminant analysis (LDA >3) scores derived from LEfSe analysis at genus level to show the different bacteria between DSS model male mice and health adult male mice that drink normal milk during lactation (mDWT-WT vs mWT-WT). (D). Linear discriminant analysis (LDA >3) scores derived from LEfSe analysis at genus level to show the different bacteria between DSS model female mice and health adult female mice that drink normal milk during lactation (fDWT-WT vs fWT-WT). n=6-8


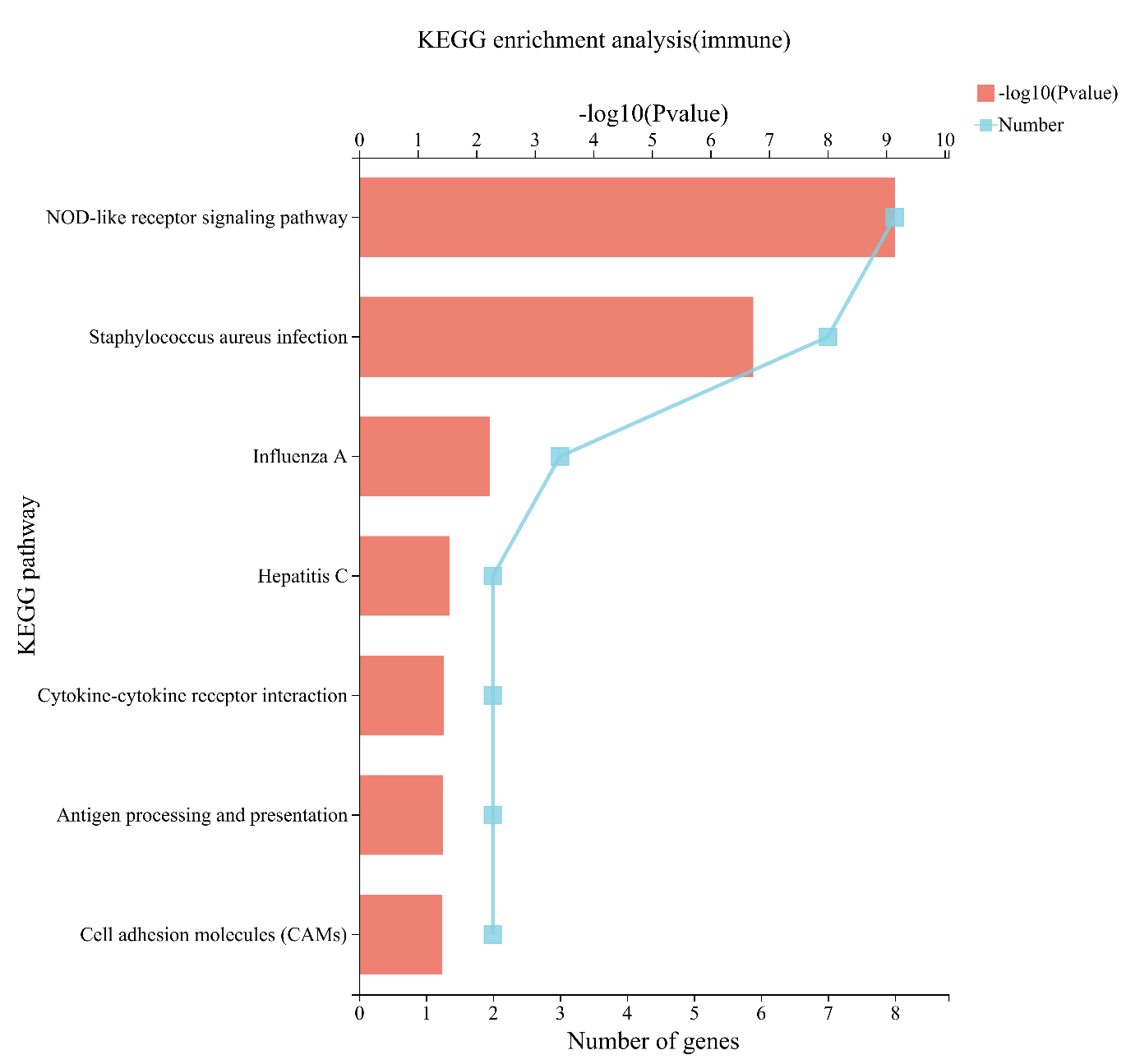


**Fig. S3**

Fig. S3. KEGG pathway enrichment analysis of immune related differential genes in 18-day-old mice small intestinal (P value＜0.05). n=3.


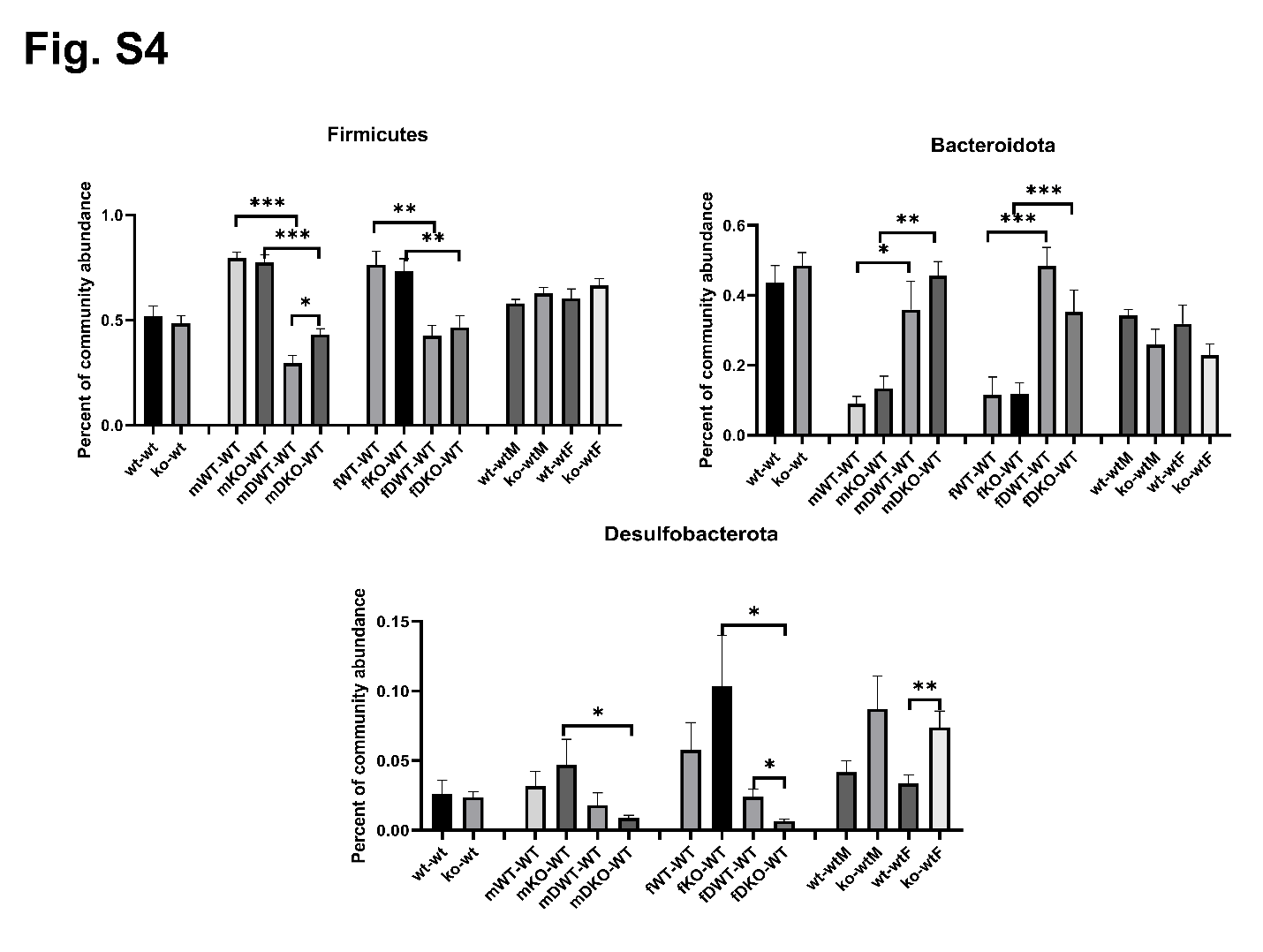
Fig. S4 The abundance of phylm Firmicutes, Bacteroidota, Desulfobacterota in 18-day-old mice, 9-week-old mice, DSS model mice and CUMS depression mice. n=6-8.


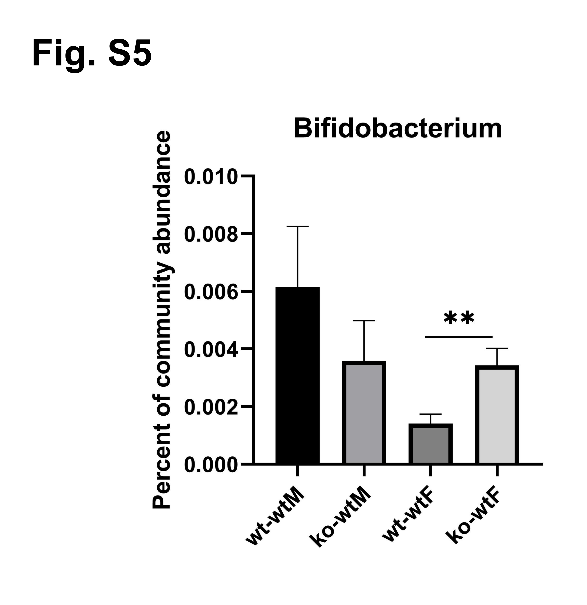
Fig. S5 The abundance of *Bifidobacterium* in CUMS mice. n=8.


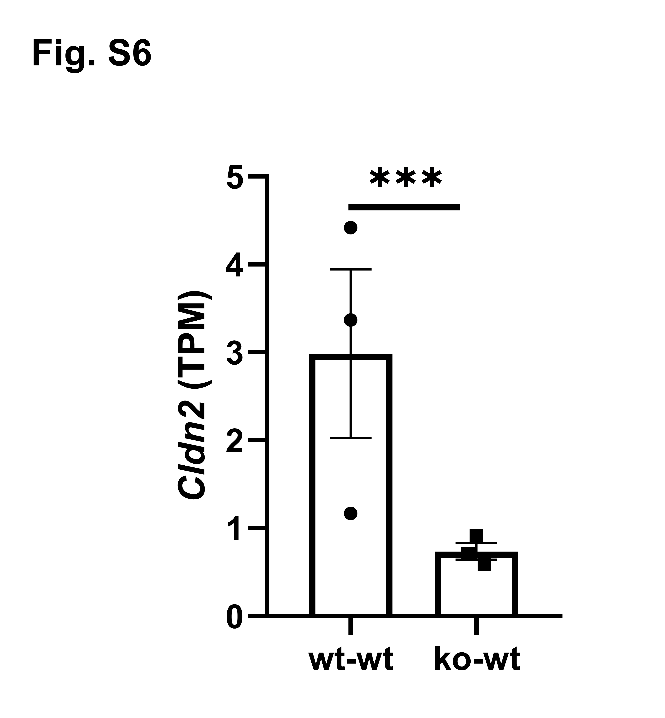


Fig. S6 Expression of *Cldn2* in the hippocampus RNAseq of 18-day-old mice. n=3.


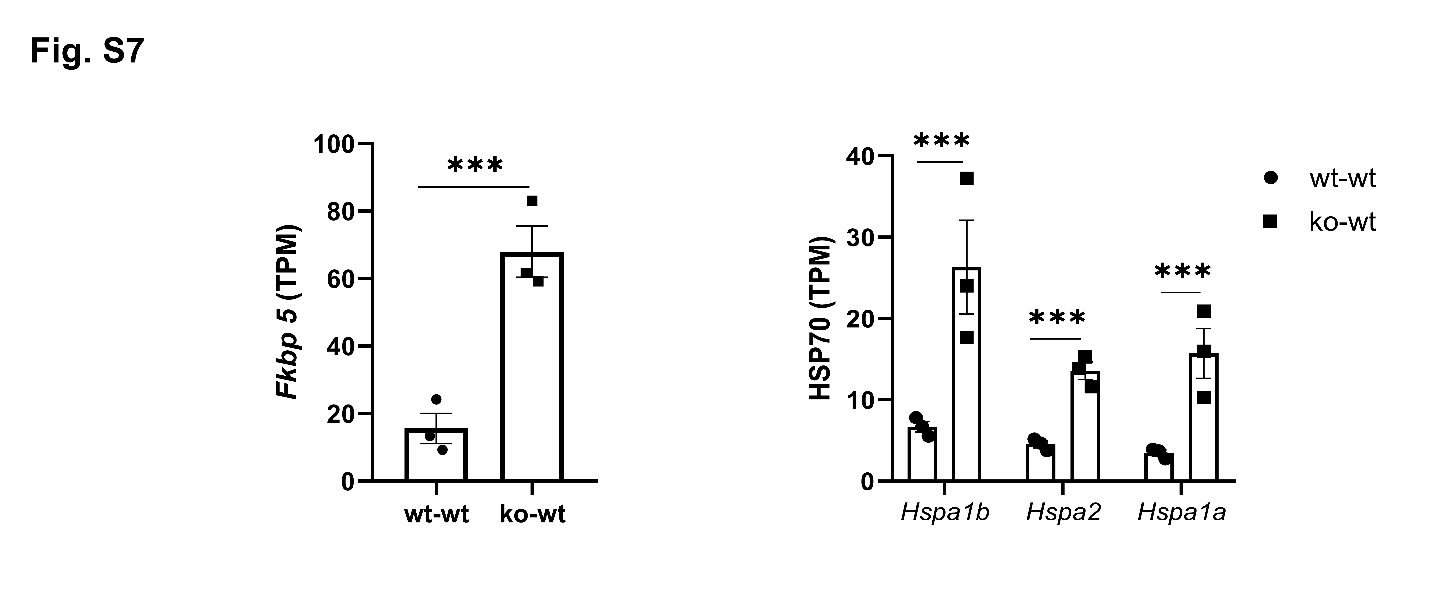


Fig. S7 Expression of genes encode FKBP5 and HSP70 in 18-day-old small intestinal RNAseq. n=3.

| **Table. S1 The R value of Fig.2L** | | | | | | | | | | |
| --- | --- | --- | --- | --- | --- | --- | --- | --- | --- | --- |
| R value | OFT | SPT | TST | FST | ACTH | CORT | IL-1β | TNF-α | LPS | BDNF |
| *Desulfovibrio* | -0.5331 | -0.9696 | 0.1378 | 0.936 | 0.8223 | 0.7166 | 0.7345 | 0.777 | 0.7221 | -0.7313 |
| *Lactobacillus* | -0.1973 | -0.3134 | 0.1289 | -0.1223 | 0.3308 | 0.4413 | 0.4356 | 0.5919 | 0.7058 | -0.5383 |
| *Enterorhabdus* | -0.3405 | -0.5093 | 0.2 | 0.07382 | 0.5116 | 0.5971 | 0.5948 | 0.7416 | 0.8337 | -0.6872 |
| *Bifidobacterium* | 0.7809 | 0.1203 | -0.9464 | 0.1918 | -0.454 | -0.5219 | -0.5059 | -0.3092 | -0.2329 | 0.4291 |
| *Eubacterium_xylanophilum_group* | -0.2269 | -0.4991 | -0.03193 | 0.8305 | 0.3581 | 0.1857 | 0.2048 | 0.1215 | -0.008491 | -0.1239 |
| *Ileibacterium* | -0.2809 | -0.2783 | 0.1599 | 0.5807 | 0.2523 | 0.1106 | 0.1221 | -0.03452 | -0.1816 | -0.01318 |
| *Dubosiella* | 0.2021 | 0.3481 | -0.01749 | -0.6986 | -0.2537 | -0.09118 | -0.107 | 0.01351 | 0.1532 | 0.01031 |
| *Alistipes* | 0.9935 | 0.681 | -0.9082 | -0.3389 | -0.889 | -0.9025 | -0.898 | -0.7716 | -0.6895 | 0.8428 |
| *Blautia* | -0.5076 | -0.7125 | 0.222 | 0.8771 | 0.6309 | 0.4843 | 0.5008 | 0.4129 | 0.2838 | -0.4246 |

| **Table. S2 The P value of Fig.2L** | | | | | | | | | | |
| --- | --- | --- | --- | --- | --- | --- | --- | --- | --- | --- |
| P value | OFT | SPT | TST | FST | ACTH | CORT | IL-1β | TNF-α | LPS | BDNF |
| *Desulfovibrio* | 0.4669 | 0.0304 | 0.8622 | 0.0640 | 0.1777 | 0.2834 | 0.2655 | 0.2230 | 0.2779 | 0.2687 |
| *Lactobacillus* | 0.8027 | 0.6866 | 0.8711 | 0.8777 | 0.6692 | 0.5587 | 0.5644 | 0.4081 | 0.2942 | 0.4617 |
| *Enterorhabdus* | 0.6595 | 0.4907 | 0.8000 | 0.9262 | 0.4884 | 0.4029 | 0.4052 | 0.2584 | 0.1663 | 0.3128 |
| *Bifidobacterium* | 0.2191 | 0.8797 | 0.0536 | 0.8082 | 0.5460 | 0.4781 | 0.4941 | 0.6908 | 0.7671 | 0.5709 |
| *Eubacterium_xylanophilum_group* | 0.7731 | 0.5009 | 0.9681 | 0.1695 | 0.6419 | 0.8143 | 0.7952 | 0.8785 | 0.9915 | 0.8761 |
| *Ileibacterium* | 0.7191 | 0.7217 | 0.8401 | 0.4193 | 0.7477 | 0.8894 | 0.8779 | 0.9655 | 0.8184 | 0.9868 |
| *Dubosiella* | 0.7979 | 0.6519 | 0.9825 | 0.3014 | 0.7463 | 0.9088 | 0.8930 | 0.9865 | 0.8468 | 0.9897 |
| *Alistipes* | 0.0065 | 0.3190 | 0.0918 | 0.6611 | 0.1110 | 0.0975 | 0.1020 | 0.2284 | 0.3105 | 0.1572 |
| *Blautia* | 0.4924 | 0.2875 | 0.7780 | 0.1229 | 0.3691 | 0.5157 | 0.4992 | 0.5871 | 0.7162 | 0.5754 |

| **Table. S3 The summary of gut microbiota results in our study** | | | | | | | | |
| --- | --- | --- | --- | --- | --- | --- | --- | --- |
| **ko-wt vs wt-wt** | | CUMS | | 18 day | 9 week | | DSS | |
|  |  | male | female |  | male | female | male | female |
| α diversity | Chao index | _ | ↓ | _ | _ | _ | _ | ↓ |
|  | Shannon index | _ | _ | ↑ | _ | _ | _ | ↓ |
| β diversity | PCOA | ** | ns | * | ns | ns | ** | *** |
| Phylum level | | ns | Desulfobacterota  Actinobacteria | ns | Patescibacteria | ns | Firmicutes  Proteobacteria | CampilobacterotaDesulfobacterota  Cyanobacteria |
| Genus level | | *Eubacterium_xylanophilum_group*  *Alistipes*  *Dubosiella*  *Lactobacillus* | *Bifidobacterium*  *Desulfovibrio*  *Enterorhabdus*  *Arenimonas*  *Lactobacillus*  *Ileibacterium* | *Bifidobacterium*  *Bilophila*  *Colidextribacter*  *Eubacterium_xylanophilum_group*  *Harryflintia*  *Lachnospiraceae_UCG-006*  *Muribaculum*  *Odoribacter*  *Oscillibacter*  *Ruminococcus*  *Blautia*  *Desulfovibrio*  *Eubacterium_brachy_group*  *Parabacteroides*  *Romboutsia*  *Turicibacter* | *Bifidobacterium*  *Lysinibacillus*  *Ileibacterium*  *Lachnospiraceae_UCG-001*  *Lactobacillus*  *Monoglobus*  *Eubacterium_xylanophilum_group*  *Kurthia*  *Enterorhabdus*  *ASF356*  *Candidatus_Saccharimonas* | *Lactococcus taiwanensis*  *Dubosiella*  *Eubacterium_brachy_group*  *Pseudogracilibacillus*  *Eubacterium_nodatum_group*  *Staphylococcus* | *Lachnospiraceae_NK4A136_group*  *Ruminococcus_torques_group*  *Odoribacter*  *Romboutsia*  *Marvinbryantia*  *Family_XIII_AD3011_group*  *Leucobacter*  *Parvibacter* | *Escherichia-Shigella*  *Enterococcus*  *Clostridium_sensu_stricto_1*  *Erysipelatoclostridium*  *Colidextribacter*  *Akkermansia*  *Ileibacterium*  *Alloprevotella*  *Desulfovibrio* |
| The red font indicates enrichment in ko-wt mice and the blue font indicates enrichment in wt-wt mice. "_", no change; "↑", up regulate; "↓", down regulate. “ns”, no significant difference; “*”, p＜0.05; “**”, p＜0.01; “***”, p＜0.001. ko-wt vs wt-wt, the mice that drink LF-free milk compared with the mice drink normal milk. | | | | | | | | |

| Table. S4 Chronic unpredictable mild stress schedule | | |
| --- | --- | --- |
| Stressors | Details | Days |
| Swimming | Mice were placed for 6 min in a cylindrical clear plastic tank (30cm high * 10cm diameter) filled with water (23±1℃) to a depth of 20cm. Immediately after the swim, mice were removed from the tank and towel-dried before being placed back in home cages. | 1, 8, 15, 22 |
| Food deprivation | Mice were subjected to 24h of food deprivation. Food was provided immediately after the end of the fasting period. | 2, 12, 18, 23 |
| Restraint | Mice were individually restraint for 4h inside 50 ml centrifuge tubes with proper holes for ventilation. | 3, 14, 21, 25 |
| Cage tilting | Cage tilting (45°) along the vertical axis for 24h. | 4, 9, 17, 24 |
| Wet cage | 200ml water in 100g bedding for 24h. Immediately after the swim, mice were removed from the tank and towel-dried before being placed back in home cages. | 5, 11, 19, 26 |
| Water deprivation | Mice were subjected to 24h of water deprivation. water was provided immediately after the end of the fasting period | 6, 10, 16, 28 |
| Tail clamping | Tail pinch 1cm apart from the end of the tail for 6min. | 7, 13, 20, 27 |
